# Determinants for Sustained Use of an Activity Tracker: Observational Study

**DOI:** 10.2196/mhealth.7311

**Published:** 2017-10-30

**Authors:** Sander Hermsen, Jonas Moons, Peter Kerkhof, Carina Wiekens, Martijn De Groot

**Affiliations:** ^1^ Institute for Communication Research Group Crossmedial Communication in the Public Domain Utrecht University of Applied Sciences Utrecht Netherlands; ^2^ Department of Communication Science Vrije Universiteit Amsterdam Netherlands; ^3^ Centre of Expertise Energy Hanze University of Applied Sciences Groningen Netherlands; ^4^ Quantified Self Institute Hanze University of Applied Sciences Groningen Netherlands

**Keywords:** mobile health, mHealth, physical activity, machine learning, habits

## Abstract

**Background:**

A lack of physical activity is considered to cause 6% of deaths globally. Feedback from wearables such as activity trackers has the potential to encourage daily physical activity. To date, little research is available on the natural development of adherence to activity trackers or on potential factors that predict which users manage to keep using their activity tracker during the first year (and thereby increasing the chance of healthy behavior change) and which users discontinue using their trackers after a short time.

**Objective:**

The aim of this study was to identify the determinants for sustained use in the first year after purchase. Specifically, we look at the relative importance of demographic and socioeconomic, psychological, health-related, goal-related, technological, user experience–related, and social predictors of feedback device use. Furthermore, this study tests the effect of these predictors on physical activity.

**Methods:**

A total of 711 participants from four urban areas in France received an activity tracker (Fitbit Zip) and gave permission to use their logged data. Participants filled out three Web-based questionnaires: at start, after 98 days, and after 232 days to measure the aforementioned determinants. Furthermore, for each participant, we collected activity data tracked by their Fitbit tracker for 320 days. We determined the relative importance of all included predictors by using Random Forest, a machine learning analysis technique.

**Results:**

The data showed a slow exponential decay in Fitbit use, with 73.9% (526/711) of participants still tracking after 100 days and 16.0% (114/711) of participants tracking after 320 days. On average, participants used the tracker for 129 days. Most important reasons to quit tracking were technical issues such as empty batteries and broken trackers or lost trackers (21.5% of all Q3 respondents, 130/601). Random Forest analysis of predictors revealed that the most influential determinants were age, user experience–related factors, mobile phone type, household type, perceived effect of the Fitbit tracker, and goal-related factors. We explore the role of those predictors that show meaningful differences in the number of days the tracker was worn.

**Conclusions:**

This study offers an overview of the natural development of the use of an activity tracker, as well as the relative importance of a range of determinants from literature. Decay is exponential but slower than may be expected from existing literature. Many factors have a small contribution to sustained use. The most important determinants are technical condition, age, user experience, and goal-related factors. This finding suggests that activity tracking is potentially beneficial for a broad range of target groups, but more attention should be paid to technical and user experience–related aspects of activity trackers.

## Introduction

### The Effect of Activity Tracker Usage on Physical Activity

One of the biggest threats to our health is physical inactivity, which is considered to cause 6% of deaths globally [1]. Too little physical activity plays a role in a range of debilitating conditions such as cardiovascular diseases, diabetes mellitus type II, chronic obstructive pulmonary disease, and some forms of cancer [2,3]. The American Heart Association endorses 10,000 steps a day or 30 min of moderate-intensity physical activity (eg, brisk walking) for at least 5 days a week as guidelines to improve health and reduce health risk [3,4]. Unfortunately, many people fail to meet these criteria [5].

Behavior change toward more physical activity might greatly benefit our health. Unfortunately, for many people, their physical activity is a deeply engrained habit [6,7]. Choosing physical activity over inactivity tends to occur outside awareness [7]. This lack of conscious scrutiny is one of the main reasons sedentary habits are difficult to change; we are not always adept in monitoring our own behavior, especially not when this behavior is executed unintentionally [8]. It is not surprising, therefore, that people tend to overestimate their physical activity [9,10]. Supporting our self-monitoring abilities by providing us with timely and relevant feedback on our behavior has proven a successful strategy to disrupt the automaticity of deeply engrained habitual behaviors such as inactivity and make them available for conscious scrutiny [11-13].

In recent years, numerous interactive and mobile technology solutions to encourage physical activity have arrived in the form of devices that are able to directly monitor our physical activity through a range of sensors. The information thus gathered can be applied by automatically providing the user of the device with behavior change techniques (BCTs) from the monitoring cluster [14]: timely feedback on their own behavior and the possibility to self-monitor behavior and its outcomes. Furthermore, dashboard applications often encourage (but hardly ever enforce) a range of secondary BCTs: goal setting, the review of behavioral goals and their outcomes, and social comparison and support.

Such activity trackers are an increasingly popular way to promote physical activity. In 2012, a survey showed that 69% of adults in the United States tracked at least one health behavior using some sort of tracking device, and 14% of US citizens owned a specialized activity tracker of some sort [15]. Of those who did track a health behavior, roughly half indicated that tracking changed their overall approach to maintaining their health (ibidem).

The effect of using activity tracker technology on physical activity is well established for a range of populations (eg, [16-19]); however, a crucial ingredient for lasting effects of behavior change interventions in general is the sustained use of the intervention [12]. Unfortunately, even though there is a growing body of research utilizing activity trackers, there is as yet little research available on sustained use of such devices. Anecdotal evidence, as well as what little evidence that is available [19], suggests activity trackers may have a poor record when it comes to sustained use, as they are easy to switch off, ignore, lose, or neglect. Furthermore, there is to date no research available that sheds light on which users manage to stick to using their activity tracker during the first year (and thereby increasing the chance of healthy behavior change) and which users stop using their trackers after a relatively short time. This paper attempts to add to our knowledge of the sustained use of activity trackers and factors that predict this sustained use.

### Potential Determinants of Tracker Use

On the basis of evidence from prior research on the effect of feedback interventions on habitual behaviors (eg, [12,20]), there is a broad range of factors that might influence sustained use and efficacy of activity trackers.

First, tracker *technology* may play a crucial role in sustained use. Trackers may be abandoned because of empty batteries, with the perceived cost of replacement too high or too cumbersome [21]. Apart from technical failures, actual or perceived characteristics of the tracker may fit user expectations. The user experience and ease of use [22,23], functionality or lack thereof [22], the possibility to upgrade toward a newer device (ibidem), aesthetics and form [24], perceived accuracy (ibidem), and perceived fit between device and self-image [23] are all reasons to either abandon the tracker or to keep using it. Furthermore, the data delivered by the tracker must fit participants’ needs (ibidem). Finally, computer literacy, or the perceived self-efficacy in using digital devices, is known to affect sustained use (eg, [25,26], higher more than lower).

*Socioeconomic status* markers such as *education* (eg, [25,27], higher more than lower) and *employment* (eg, [28,29], higher more than lower), *age* [25,30,31], older more than younger) and *gender* (eg, [25,27,32], women more than men) are known to influence sustained use, as are *psychological traits* such as inhibitory strength and the capacity for self-regulation [33-35].

Personal *health-related factors* may very well influence the sustained use of the activity tracker; poor health decreases perceived self-efficacy [36,37], which is known to influence sustained use [38]. Low mood, stress, sleep disturbances, and other markers of mental health, are also known to decrease sustained use [30,39].

*Goal-setting* is generally seen as a promising strategy to increase the use of physical activity interventions [40-42]. Strong, clear goals and *motivation* to fulfil these goals [25,30,31] increase the chance of sustained tracker use. *Achieving* these goals, or at least displaying a performance level that could lead to achieving previously-set goals, can provide a further boost to initial motivation and perceived self-efficacy, increasing the chances of sustained tracker use. However, the fulfilment of a set goal may also lead to device abandonment, because users feel they no longer need the tracker [43].

Furthermore, behavior change theories (eg, social cognitive theory [44] and control theory [35]) suggest that behavior change is most likely if feedback is not delivered on its own but *embedded* in larger interventions with clear target behaviors and action plans. Combined use of the activity tracker with other health apps, participation in a therapeutic regime, and use of the app and Web-based platform that accompany the activity tracker may be seen as an operationalization of this concept of integration. Overall, we expect users with strong goals and high integration of their tracking behavior in other health-related practices to have a higher chance of sustained tracker use, especially when these users manage to achieve their performance goals.

*Feedback properties* such as timing, duration, frequency and sensory modality (cf [20]), and *user experience* (eg, [45]) are known to influence the efficacy of the feedback intervention, both directly and through perceived usability and agreeableness. Similarly, feedback properties [46] and user experience-related factors are known to affect the uptake and sustained use of physical activity trackers [47]. We expect users with greater liking of the tracker and its accompanying online tools to have a higher chance of sustained tracker use.

Activity tracking is often social and collaborative instead of individual and personal [24,48,49]. *Social interaction* is known to improve adherence to physical activity interventions in general [50]. We therefore expect users that share their tracking data with peers or relatives to have a higher chance of sustained tracker use.

### Sample Size and Duration in Previous Research on Activity Trackers

Current research into determinants of activity tracker use typically makes use of small test populations, ranging from 7 to 31 participants (eg, [23,24,48,49,51-53]), which limits the possibilities to reliably investigate quantitative measures of determinants of device use. When larger samples have been tested (eg, [22], n=1561 and [39], n=256), only a small number of determinants were included. Furthermore, adherence studies generally covered only a very short period, that is, 2 months or less (eg, [23,51-53]). Only one study ([54]) tested sustained use over a period of up to 10 months. However, this study did not evaluate potential determinants for adherence.

This study attempts to contribute to bridging this knowledge gap by looking into factors predicting sustained use in the first year after purchase. Specifically, we look at demographic and socioeconomic, psychological, health-related, goal-related, technological, user experience–related, and social predictors of feedback device use and their predictive power in determining which participant is most likely to continue using the device.

## Methods

### Study Design

This study was initiated by IDS Santé Inc (Paris, France), a full-service communication agency aimed at the health sector and specializing in prevention and health education and executed from June 2013 until winter 2014 as a project called “MySantéMobile.” A total number of 1000 participants were recruited in France via a (free) newspaper from four French cities (Bordeaux, Lille, Montpellier, and Lyon). Each participant received an activity tracker and was requested by email to fill in three Web-based questionnaires (June 2013, August 2013, and January 2014). After completion of the study, the full raw dataset was transferred for independent and retrospective analysis to the authors of this paper.

To establish which set of the included predictors best explains the use and nonuse of this activity tracker in the dataset, we adopted the Random Forest method, a machine learning approach [55]. This approach enables identification of predictors that explain large portions of variance while minimizing the risk of overfitting, which is likely to occur when performing a regression analysis with a large set of predictors [56]. Furthermore, this approach is also capable of detecting nonlinear relationships and higher-order interactions between predictors.

### Activity Tracker

The activity monitor used in this study, the Fitbit Zip, is a small (2.9 cm x 3.6 cm x 1 cm) consumer device that tracks activity through counting steps. The Zip is worn as a clip-on device on the waist or elsewhere where it can be easily clipped onto clothing. On the device screen, the Zip displays the number of steps taken on the current day, and, after pressing a button on the device, displays the distance covered on the current day, active minutes, the time, an approximation of calorie expenditure, and feedback in the form of a happy, neutral, or unhappy *smiley*. Research [57,58] shows that the reliability and validity of the Fitbit Zip activity monitor is high, with little error in the number of registered steps, both in laboratory conditions and in daily life.

### Participants

#### Recruitment

Participants were recruited through a newspaper article, published on the 14th of May 2013, in free newspapers in France. 1000 participants were selected using the following inclusion criteria: living in one of the four eligible cities (Montpellier, Lyon, Lille, and Bordeaux); at least 18 years of age; and owning a smartphone or computer compatible with Fitbit. Of those 1000, 929 received a Fitbit Zip activity tracker and took part in the study.

#### Data Acquisition

In the first week of June (2013), all eligible participants were invited to fill out a Web-based questionnaire by email. This questionnaire was presented through the LimeSurvey platform and covered sociodemographics, device usage, tablet/phone brand, self-reported tracker use, use of other health apps and devices, health, exercise, and diet. All questionnaires used in this study are available in Multimedia Appendix 1. Approximately two weeks after filling in this questionnaire, the participant received their Fitbit Zip tracker by mail. Participants received their Fitbit Zip tracker free of charge.

Upon dispatch of the Fitbit trackers, participants received an email giving them instructions on how to install and use the Fitbit, how to synchronize data and how to authorize MySantéMobile in acquiring their data through the Fitbit API. Instructions were also provided on the MySantéMobile website. Participants then had to give permission to MySantéMobile to read their activity data through the Fitbit API. Participants who did not give permission received reminder phone calls and emails.

A second questionnaire was sent out by email on 23 August 2013 (after 98 days). The third questionnaire was also sent out by email, on 7 January 2014 (232 days). Participants who did not fill out the questionnaire received a reminder email after two weeks. Participants received no incentive other than a free activity tracker. At the end of the data acquisition period, all participants received an overview of the study results.

#### Participant Selection

Since the selected analysis method does not allow missing values, only data from those participants who completed their questionnaires could be used. Of the 929 participants originally approached to take part in the study, 711 participants (76.5%) completed the first questionnaire and gave permission to MySantéMobile to read their activity data through the Fitbit API (Textbox 1). Data collection using the Fitbit took place from 20 June 2013 to 13 May 2014 (327 days). Of this group of 711 participants, a total number of 575 participants (80.8%) completed the second questionnaire (August 2013) and 542 participants (76.2%) completed both the second and the final questionnaire (January 2014).

**Participant characteristics at Q1.**

- Gender
  • 330 female, 381 male
- Age
  • < 25: 133, 26-35: 444, 36-45: 182, 46-55: 124, 56-65: 49, >65: 4
- Marital status
  • Single: 240, Couple: 332, Single parent: 38, Family: 272, Other: 54
- Profession
  • Cadre (management): 456, Intermédiaire (middle management): 91, Employé (employee): 251, Artisan (craftsperson): 51, Ouvrier (worker): 12, Retraité (retired): 19, Sans (without): 56
- Education
  • Bac: 106, Bac+2: 371, Bac+5: 406, CAP/BEP: 38, Brevet des Colleges: 11, None: 2

### Measures

The total number of days on which the device was worn was used as the primary outcome measure (adherence to using the wearable for self-tracking). We only had access to data that were synchronized with a personal computer or mobile app. However, the Fitbit Zip stores steps data for 30 days, therefore, we assume most active users will synchronize their data within this time window. For ease of interpretation, we will speak of “using” or “wearing” the Fitbit. However, note that our measure may somewhat underestimate the number of days the Fitbit was worn.

Furthermore, we calculated the average amount of steps taken by each participant on those days the tracker was used.

### Questionnaires and Item Selection

Three questionnaires (Q1, June 2013; Q2, August 2013; Q3, January 2014) were sent out to the participants. A complete overview of all three questionnaires, with the exact questions (translated into English), and the response scales used for each question, is available as Multimedia Appendix 1.

From these questionnaires, we selected for our analysis those items that (1) matched the potential determinants for sustained use of the tracker outlined in the introduction of this paper, and (2) met with our requirements for item validity.

On the basis of our analysis of potential determinants for sustained use, we included the following items from the questionnaires in our analysis:

1. Demographical and socioeconomic factors: age, gender, place of residence, household size and household composition, profession, and education (all in questionnaire 1 (Q1).
2. Psychological factors: general mood (all questionnaires); specific scores on affective situation (sadness, gaiety), stress (calmness, stressfulness), energy (energy level, tiredness), and sleep quality (all in all questionnaires); big five personality traits (openness to experience, conscientiousness, extraversion, agreeableness, and neuroticism; plus, rebelliousness, health-mindedness, and independence (all in Q3).
3. Technological factors: synching platform type (smartphone, tablet, computer), operating system—iOS or Android (all in Q1), use of other health applications (Q1), experience with technology (Q3).
4. User experience: perceived utility, enjoyableness, intrusiveness, modernity, fun, reliability, simplicity, inconvenience, correspondence to needs, beauty, robustness, and cumbersomeness of the activity tracker (all in Q2); exactness, detail, clarity, credibility, confidence, insight, perceived efficacy (all in Q3).
5. Health-related factors: body mass index (all questionnaires), smoking (Q1), pregnancy (Q1), diet (Q1), medical treatment status (Q1), activity in sports (Q1), and sports together with others (Q1).
6. Predefined participant goals and perceived goal achievement: increasing activity, improving sleep, quitting smoking, diagnosing or improving diet, diagnosing behaviors, losing weight, and improving stamina (all in Q1); for each goal, the perceived achievement of the goal was measured (Q2 and Q3).
7. Social factors: whether participants talked about the tracker sharing use with family, friends, colleagues, teams and clubs; sharing data on the Internet through social media, blogs, Twitter, websites, forums, and mailing lists (Q2 and Q3).

### Questionnaire Validity

Because of the history of this study, which started as groundwork for a publicity campaign for a communications agency, the questionnaires used in this study have not been constructed in such a way that meets the current standards for validity. To evaluate the validity of the three questionnaires used in this study and to determine which items were of high enough standard to include in our analysis, we compared each question with current, well-validated standard approaches in scientific literature. The complete result of this analysis is included in Multimedia Appendix 1. For each item, under “remarks,” the validity evaluation is listed. Generally, our evaluation showed that the greater part of the questionnaire items survives rigid scrutiny and satisfies scientific criteria. However, the validity of four items, one item on digital proficiency and three items on psychological traits (rebelliousness, independence, and health-mindedness) could not be satisfactorily assessed. Results for these items should be used with caution.

The greater part of the questionnaire consisted of single-item measures. Single-item measures can be eminently usable (sometimes even more so than multiple item measures) when the attribute (eg, attitude, frequency) is concrete and singular (ie, not consist of multiple facets) and when the object of the item (eg, brand, product) is concrete [59]. For most of the items, this is the case; see Multimedia Appendix 1 for an overview. Three exceptions occurred, which are as follows: items regarding emotional well-being, items regarding psychological traits, and items regarding user experience.

The first group of items that do not have a concrete object are about emotional well-being. However, single-item assessments of emotional well-being are often used in large-scale surveys (see [60] for an overview) and have been shown to perform quite well compared with multiple-item scales (eg, [61]). We can therefore probably conclude that these single-item self-report measures are a sufficiently valid measure for the purpose of this paper.

The second group of items that do not have a concrete object concern psychological traits. In Questionnaire 3, the big five personality traits (openness to experience, conscientiousness, extraversion, agreeableness, and neuroticism) are measured using the French translation of the TIPI questionnaire [62]. This questionnaire has been well validated.

A third group of items address the user experience of the Fitbit. User experience can be defined as *a person’s perceptions and responses that result from the use and/or anticipated use of a product, system, or service* [63]. In recent years, approximately a hundred different measures have been developed [64]. User experience evaluations generally consist of questions addressing some (but never all) of the following concepts: timeliness, adaptability, comfort, opacity, efficiency, immersion, intuitiveness, ease of use, usefulness, interaction, controllability, clearness, completeness, identity, novelty, originality, fun, stimulation, valence, connectedness, attractiveness, beauty, and trust [65]. Currently, no questionnaires or other measures exist that address all of the aforementioned concepts. In this study, comfort, ease of use, novelty, fun, valence, attractiveness, trust, and invasiveness have been measured.

Concepts regarding user experience can be subdivided in three categories [66]: pragmatic qualities (usability-oriented; first 13 concepts [timeliness-completeness]), hedonic qualities (identity-connectedness), and general concepts, mainly focused on attraction. Principal component analysis showed that the response patterns for the 22 user experiences-related items asked in questionnaires 2 and 3 justified the construction of three conceptual factors. The first factor was the valence of the activity tracker, which was formed by the following 12 items: usefulness (practicality), niceness, modernity, amusingness, credibility, ease of use, level of answering to needs, beauty, robustness, intrusiveness, embarrassment, and nuisance. This factor corresponds with the hedonic quality in [66]. The second factor was the preciseness of the activity tracker, which was formed by the following 4 items: exactness, level of detail, clarity, and credibility. This factor corresponds with the pragmatic qualities in [66]. The third and final factor was perceived efficacy of the activity tracker, which was constructed by averaging 3 items on perceived efficacy of the tracker, namely activity increase, health changes, and well-being. This third factor does not correspond with hedonic nor pragmatic qualities but has to do with the perceived effectiveness of the activity tracker. The results of the principal component analysis are reported in Multimedia Appendix 2.

On the basis of our analysis, some items were left out of our analysis; see Multimedia Appendix 1 under “left out” for each questionnaire. Four items, of which the validity could not be determined satisfactorily, were nevertheless included in the analysis. One item concerned technological aptitude, and three further items concerned the psychological traits: rebellion, independence, and health-mindedness. We advise to treat the results of these items with caution.

### Statistical Analysis

We determined the relative importance of all included predictors by using Random Forest, an analysis technique based on recursive partitioning [55,56]. Random Forest is an ensemble method that makes use of a large number of decision trees, strengthened by “bootstrap aggregating”: drawing random samples from the original dataset with replacement. For each of the bootstrap samples that are drawn, a decision tree is constructed. At each branch of the tree, a random selection of the predictor variables is considered. The variable that produces the best split (ie, most informative and offering the largest contrast) is used to divide the cases over two daughter nodes.

To predict the outcome variable for a specific case, Random Forest uses the predictions of all trees to arrive at an “ensemble” prediction (in the case of regression, it averages the prediction of all trees). To evaluate the performance of the Random Forest model, we can test each tree on those cases that fell outside its bootstrapped sample and thus, were not used to grow the tree. This produces an “out-of-bag” error rate, which is a good approximation of the test error.

Random Forest analysis can produce a list of predictors, sorted by relative importance. This is done by calculating the mean squared error (MSE) and looking at the relative increase of the MSE when the values of a predictor are permuted across cases. Permuting the predictors retains frequency information but destroys the association between the predictor and the outcome variable. If the variable is important for the Random Forest model, we would expect its predictions to deteriorate and the MSE to go up. Thus, the relative increase in MSE is used to determine an importance ranking of predictors, sorted from greatest to least increase in MSE.

Random Forest modeling has the benefit of being able to deal with large numbers of predictor variables with complex interactions, especially in situations with relatively few cases relative to the number of predictors. Furthermore, Random Forest is capable of detecting nonlinear relations between independent and dependent variables. Random Forest analysis methods have recently been applied successfully in genetics, clinical medicine, bioinformatics, and the social sciences (see [56,67] for examples).

The predictor variables for the Random Forest analysis were taken from the responses to the questionnaires. All parameter settings, source code, and data files for the Random Forest analysis are available through the Open Science Foundation.

We used R 3.3 for analysis [68] and the R package “randomForest” [69] for Random Forest modeling.

## Results

### Fitbit Use

**Figure 1:**
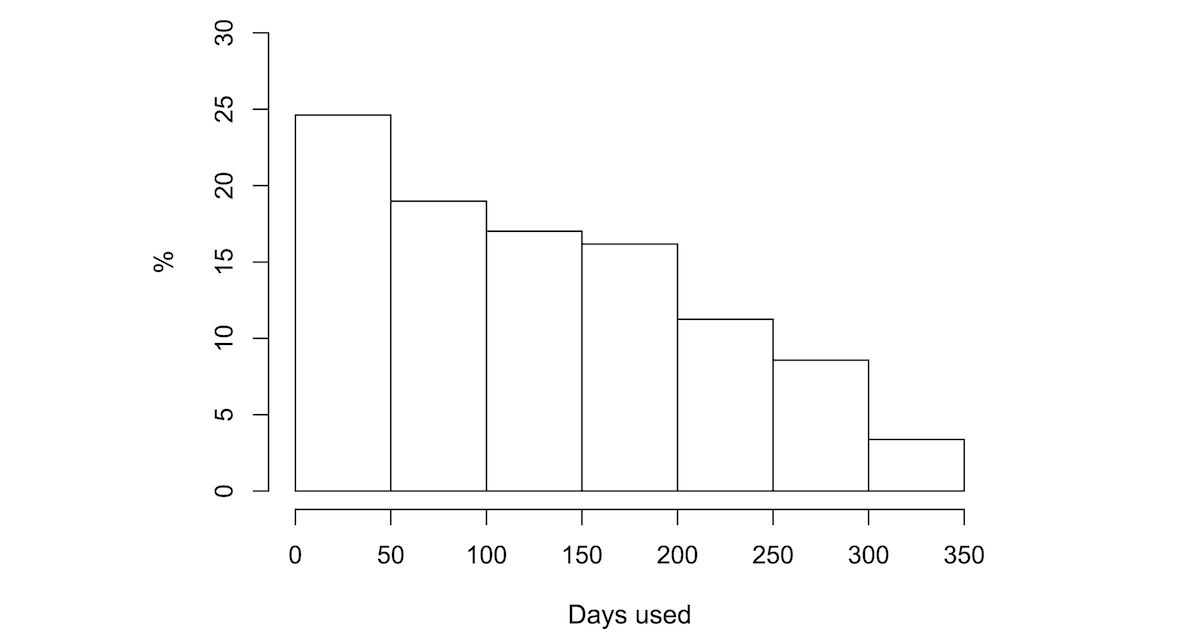
Distribution of participants’ total number of days of activity tracker use.

The mean number of days that participants used their Fitbits was 129.3 (nonconsecutive) days (standard deviation [SD]=88.5; median=122). Figure 1 shows the distribution of total days of use.

**Figure 2:**
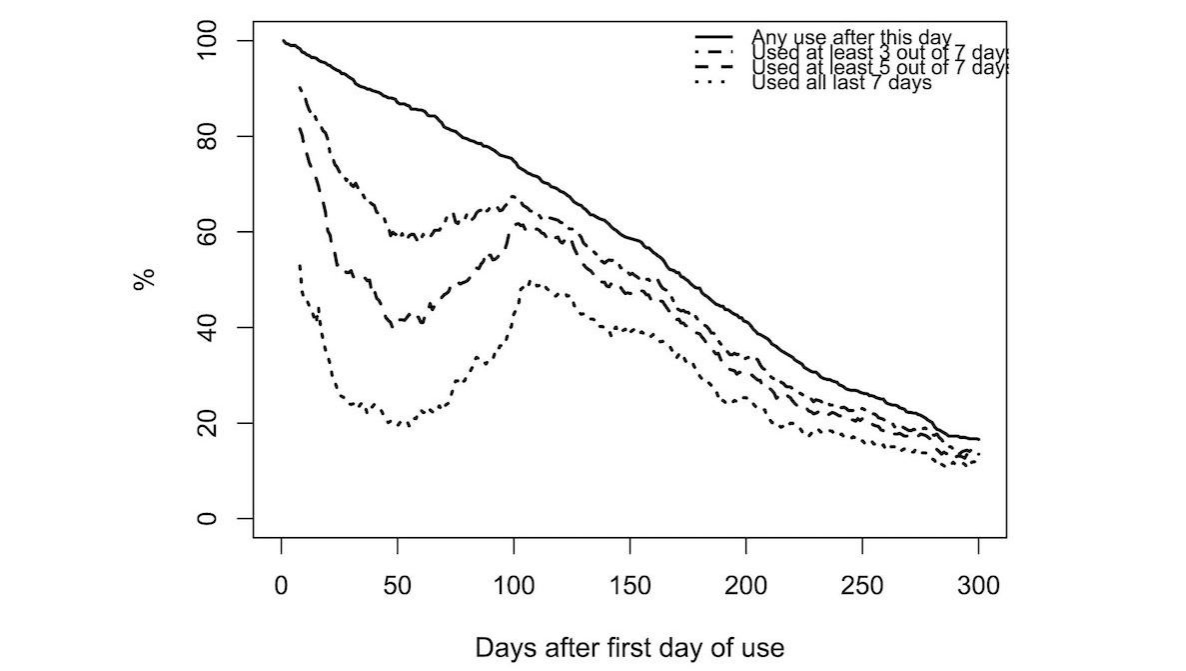
Usage decline over time. The horizontal axis shows the number of days since the first day of use. The percentage of participants who used the activity tracker for any number of days after a particular day is indicated with a solid line. The other lines indicate habitual use: the percentage of participants who used the tracker for at least 3, 5, and 7 days in the preceding 7 days. Note that this includes participants who stop using the tracker and later start using it again. The early dip in use is due to the summer holiday.

A graphical overview of usage over time is shown in Figure 2. As some users only started to wear the Fitbit after weeks or even months, the number on the x-axis refers to the number of days since the first day the device was used, rather than from the start of the study. The figure indicates both the percentage of participants who used the device for any length of time after the indicated day and habitual use (defined as 3, 5, and 7 days worn out of the last 7 days). The decline during the first 50 days coincides for most users with the French holiday season (July-August). The peak shortly after 100 days coincides with Q2 being sent out (again, for most users).

The pattern of (nonhabitual) usage decline is roughly linear. A linear regression with time as the independent variable shows a decline of 2.0 percentage points per week from day 1 to day 300. In other words, every week, 2% (14) of the participants at start stopped using the tracker entirely. As the base of users is shrinking, this means that the proportion of participants who stopped using the tracker increases over time. After 175 days (5.7 months), 50% of users have stopped wearing their tracker.

Habitual use seems to follow a pattern of slow exponential decay. An exponential model shows that the proportion of users wearing the activity tracker 5 or more days per week declines 5.7% per week, as calculated from the peak after the summer holiday dip (from day 102 to day 300).

**Figure 3:**
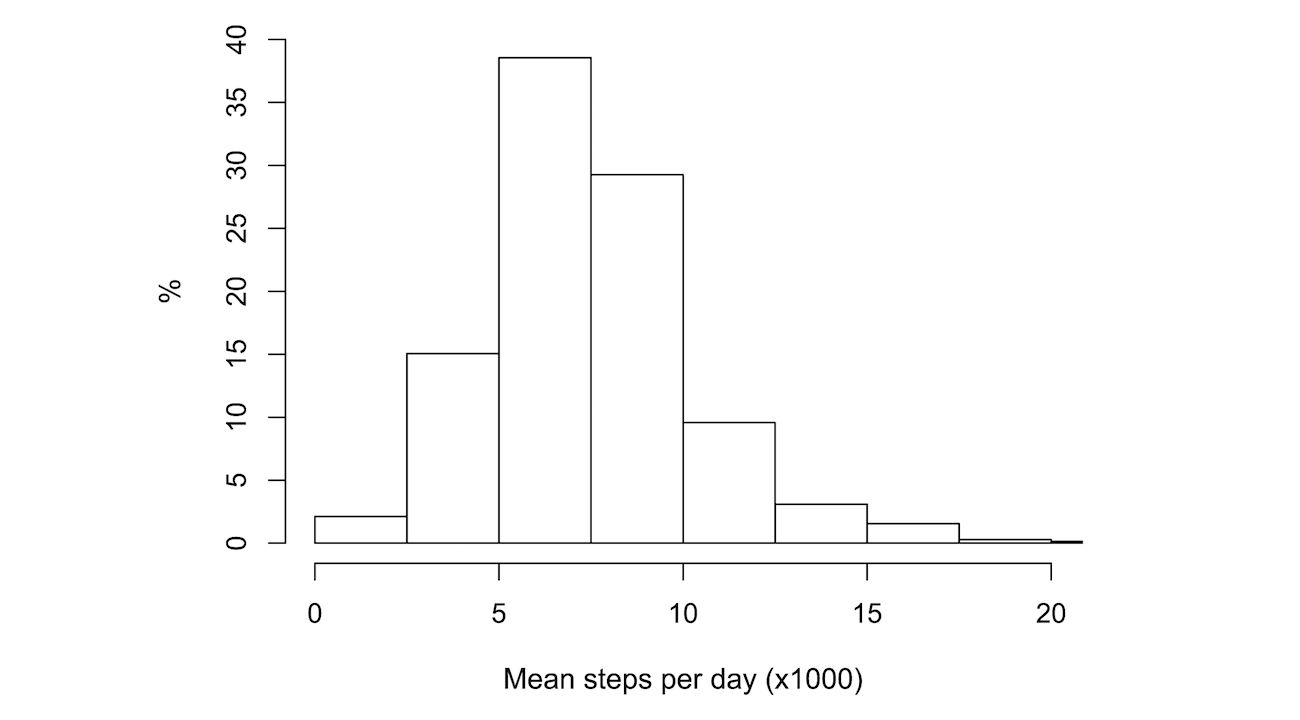
Distribution of participants’ mean number of daily steps.

On average, participants took 7492 steps (SD 3012) per synced day (ie, day on which they wore their tracker). The median number of steps was 7107, with an interquartile range of 3462. A plot of the distribution of the number of steps taken is provided in Figure 3.

An overview of the correlations between the number of days on which the tracker was used, mean number of steps, and a range of self-report measures on personal health are displayed in Table 1. The number of days the activity tracker was used significantly predicted mean steps per day: *b*=9.43, *t*_709_=32.60, and *P*<.001. A significant proportion of the variance was explained: *R*^2^=.08, *F*_1,709_=8.92, and *P*<.001. An exploratory analysis of correlations with measures on personal health showed generally weak to negligible associations for both days used and mean steps per day. Strongest associations were between the number of days used and self-reported general health (*r=*.16) and between days used and physical shape (*r*=.12).

**Table 1:** Correlations (Spearman *r,* unless marked with b: Pearson *r*) between steps taken, days worn, and health measures. All measures are from Q3 unless otherwise noted.

Health measure	Mean steps	Days used
Mean steps		.28^a,b^
Days used	.28^a,b^	
Self-reported weight change (kg, Q3-Q1)	−.05^a^	−.04^a^
Self-reported effect tracker on weight	.04	.10^d^
Self-reported effect tracker on activity	.02	.04
Self-reported effect tracker on sleep quality	.05	.08
Self-reported effect tracker on smoking	−.03	−.07
Self-reported effect tracker on healthy eating	.04	.07
Self-reported effect tracker on general health	.04	.16^b^
Self-reported effect tracker on physical shape	.07	.12^c^
Self-reported tiredness	−.04	−.06
Self-reported happiness	.04	.07
Self-reported stress	−.04	.01
Trend in self-reported tiredness (Q3-Q1)	.07	.03
Trend in self-reported happiness (Q3-Q1)	.04	−.01
Trend in self-reported stress (Q3-Q1)	.03	−.01

^a^Pearson *r*.

^b^Significance at *P*<.001.

^c^Significance at *P*<.01.

^d^Significance at *P*<.05.

**Table 2:** Reasons for not wearing the Fitbit.

Reason to not wear	Q2 (98 days)			Q3 (232 days)		
	Count	% of total reasons	% of total respondents Q2 (639)	Count	% of total reasons	% of total respondents Q3 (601)
Technical failure or difficulty	20	50.0	3.1	106	56.7	17.5
Lost the device	9	22.5	1.4	24	12.8	4.0
Forgot to wear	2	5.0	0.3	24	12.8	4.0
Had no use for device or no motivation	0	0.0	0.0	16	8.6	2.6
Health issues	1	2.5	0.2	7	3.7	1.2
Used other device	0	0.0	0.0	2	1.1	0.3
Was on holiday	7	17.5	1.1	2	1.1	0.3
Did not start yet	1	2.5	0.2	0	0.0	0.0
Other	0	0.0	0.0	6	3.2	1.0
Total	40	100	6.3	187	100	30.8

### Reasons for No Longer Using the Tracker

In both Q2 and Q3, participants were asked how many of the last 30 days they wore their Fitbit. If the answer was “fewer than 5,” they were asked additionally why they did not wear their tracker (more often) in an open-ended question. The responses were categorized and can be found in Table 2.

The results from Q2 (sent after 98 days) indicated that 40 participants (6.3% of all respondents) used the Fitbit fewer than 5 days in the last month. The primary reason for not using the device, given by half of those indicating low or nonuse, was technical failure or other technical problems, including empty batteries. Other reasons included losing the device or being on a holiday. Technical problems were also the main reason given in Q3 (sent after 232 days), with 17.5% of all respondents reporting this issue.

### Factors Associated With Usage

We used the Random Forest method to investigate which predictors are associated with continued use of the activity tracker. The total number of days on which the device was worn was used as the outcome variable. As many participants did not respond to all three questionnaires, with those who stopped using their Fitbit less likely to fill in questionnaires 2 and 3, we decided to construct two different models, corresponding to two different groups of participants: (1) participants completing Q1 and (2) participants completing all three questionnaires (Q1 to Q3). For the latter model, we analyzed only those participants who did not state technical malfunction of any kind as a reason to quit.

In the first model, the data from those 586 participants who completed Q1, gave permission to use their tracker data, and stated neither technical issues nor lost trackers as a reason to no longer track, were entered. Some predictors were adjusted or recalculated. A complete overview of all questionnaires, the exact questions, the response scales, and any recalculations or adjustments is available in Multimedia Appendix 1.

**Figure 4:**
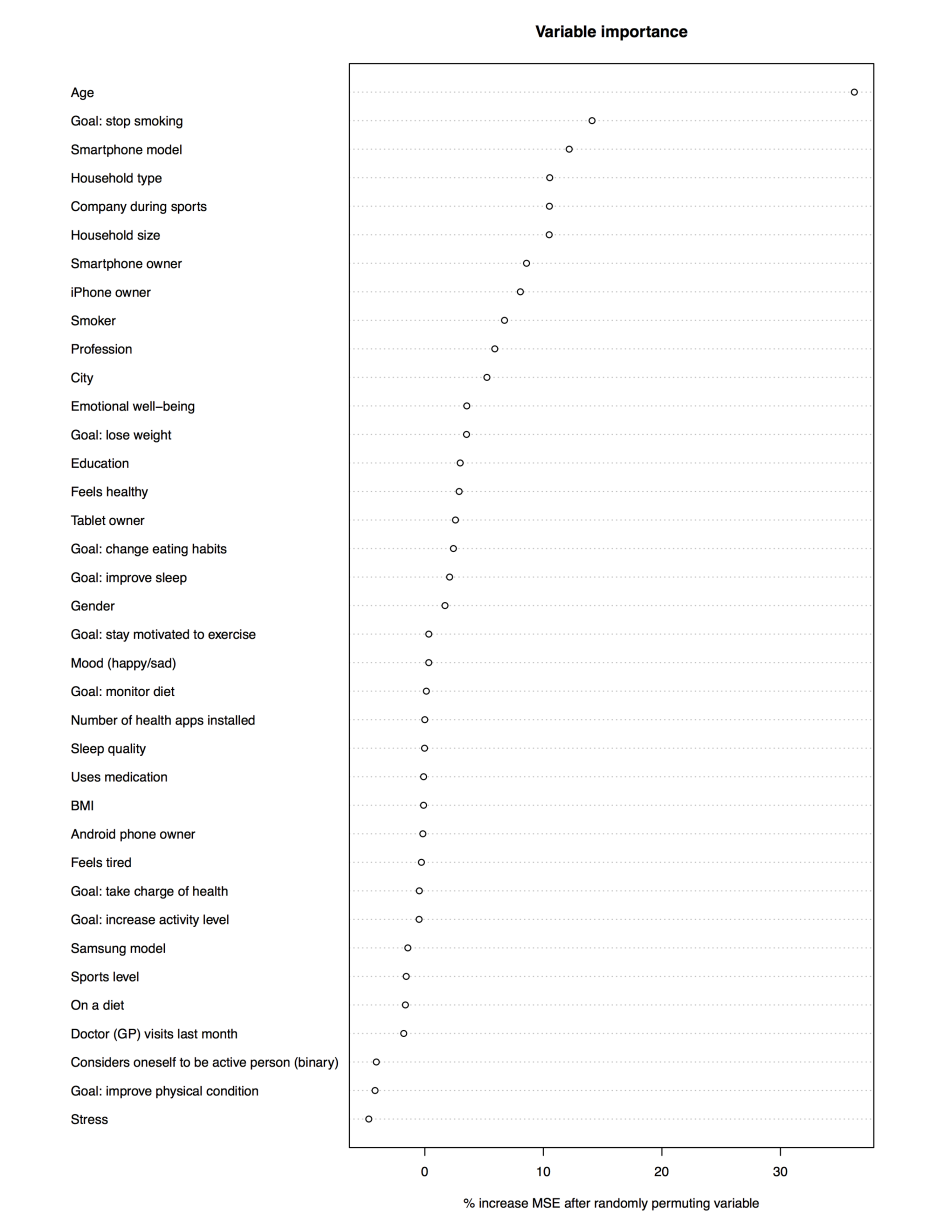
Plot of relative importance of predictors of sustained use in questionnaire 1 (Q1); BMI: body mass index, MSE: mean squared error.

Figure 4 shows the relative impact of each predictor variable in Q1 on the amount of variance explained, expressed as the relative increase in MSE when the predictor is randomly permuted across participants. The Random Forest model with all Q1 predictors included explains 8.29% of variance. Only those predictors whose increase in MSE is above zero are displayed because decreases can be safely attributed to noise.

**Figure 5:**
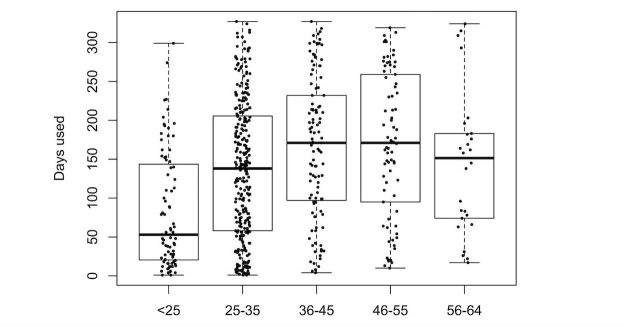
Boxplots of the distributions of Age levels. Older participants have longer sustained use.

**Figure 6:**
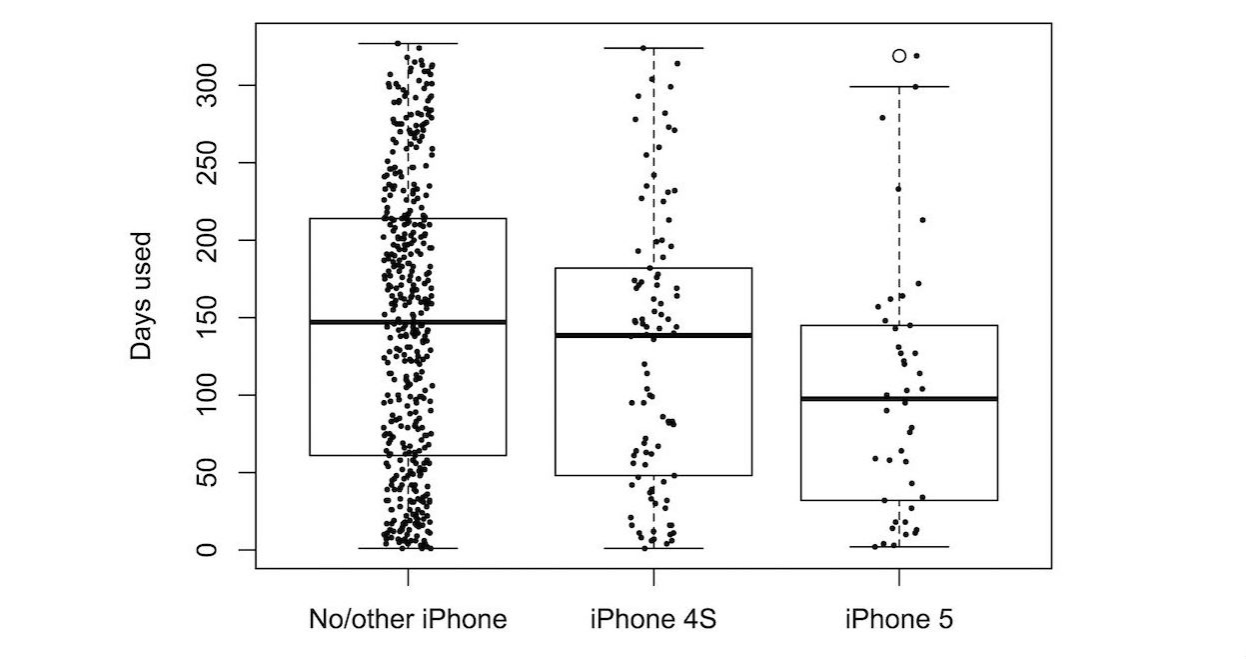
Boxplots of the distributions of iPhone type levels. Holders of iPhones show less sustained use than those of other smartphones.

**Figure 7:**
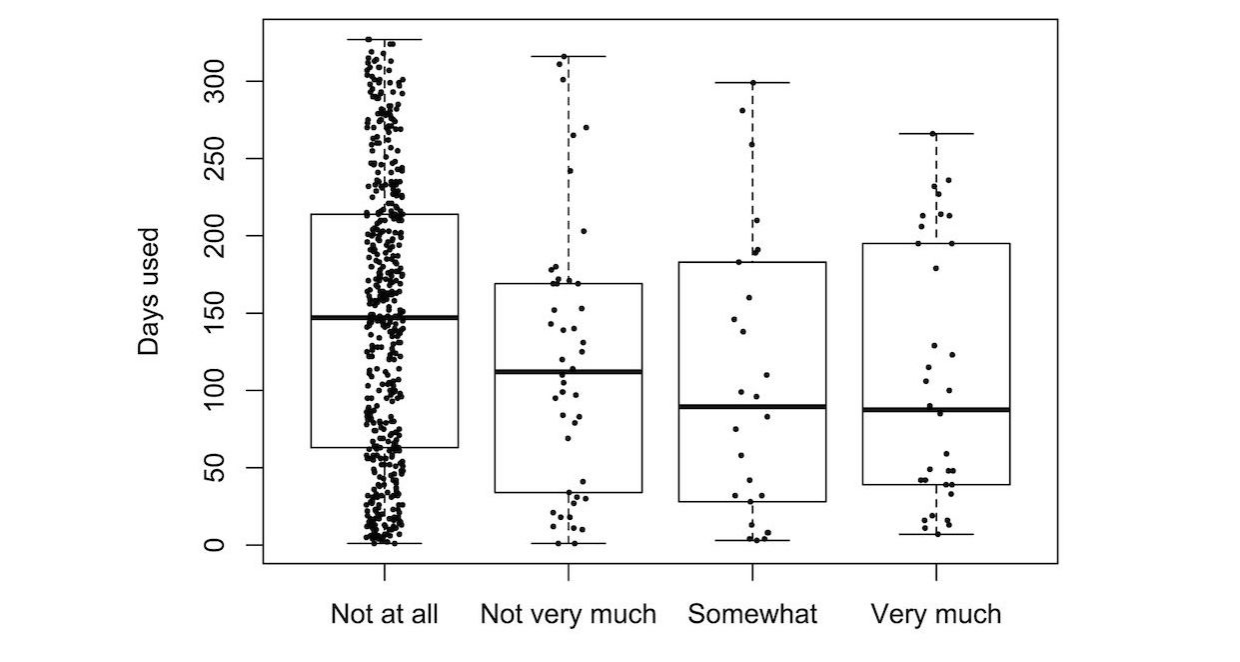
Boxplots of the distributions of Having the goal to quit smoking. Those not wanting to quit smoking (including non-smokers) have longer sustained use than those who do.

**Figure 8:**
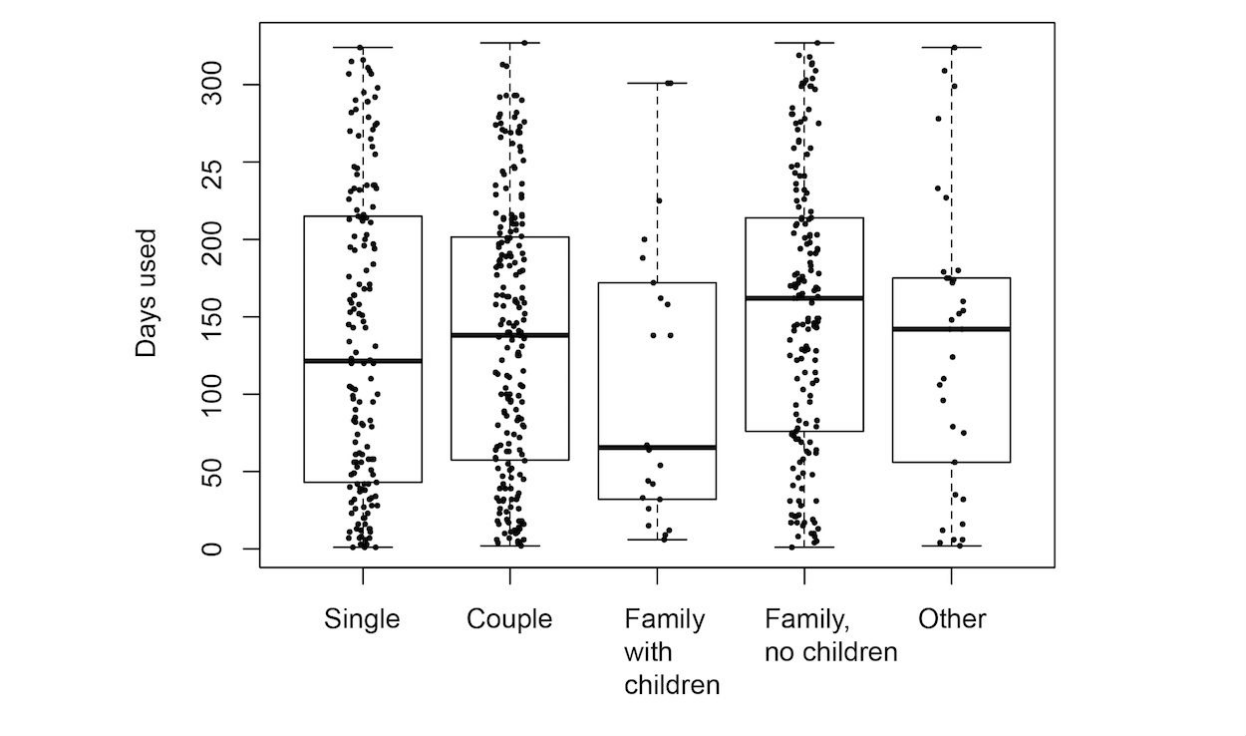
Boxplots of the distributions of Household type. Single parents show shorter sustained use than other household types.

**Figure 9:**
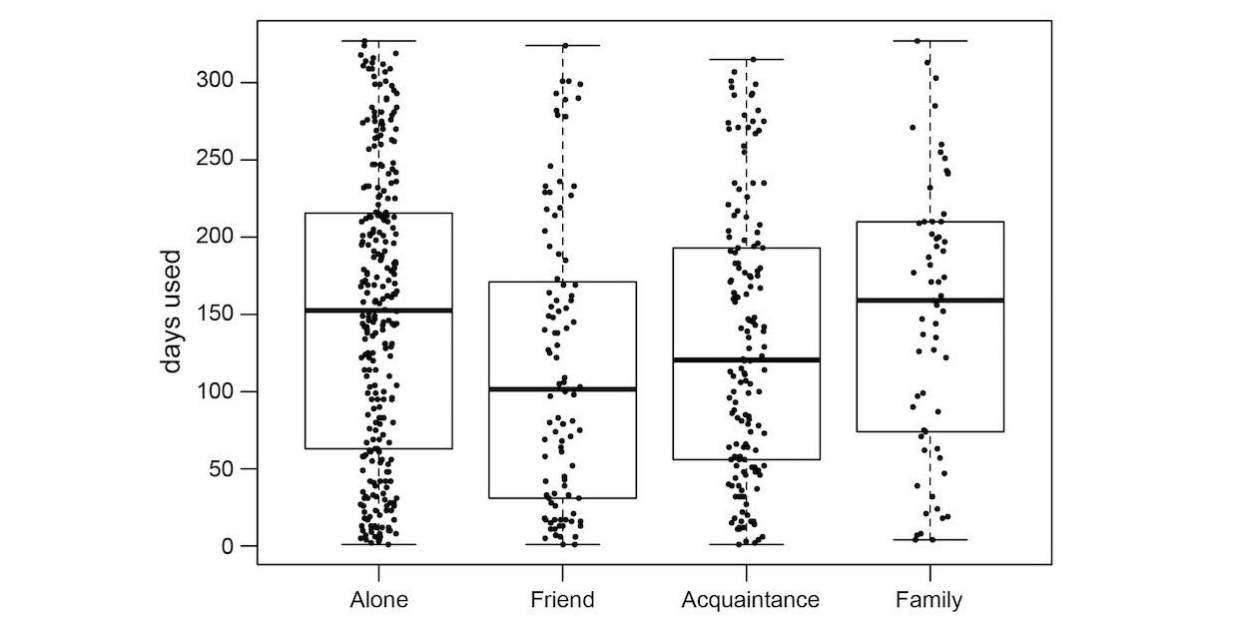
Boxplots of the distributions of Sports in company of others. Those who practice individual sports or with relatives, have longer sustained use of the tracker than those who participate in sports with friends or acquaintances.

We explored the effect of the different predictors on the number of days the participants wore their tracker. Figures 5-9 provide boxplot representations of the distributions for the marginal means (with all other factors kept constant) of the different levels of each predictor. Only those predictors of which the differences in marginal means implies a meaningful difference in real life (>1 day) are included: Age, goal to quit smoking, iPhone type, sports activities in the company of others, household type, household size, having a smartphone, having an iOS-based-smartphone, profession, and smoking.

In the second model, data from 397 participants who completed all three questionnaires (Q1 to Q3) and who did not state technological malfunction as a reason to stop tracking, were entered. Once again, some predictors were adjusted or recalculated. A complete overview of all questionnaires, the exact questions, the response scales, and any recalculations or adjustments is available in Multimedia Appendix 1.

Figure 6 shows the relative impact of each predictor variable in the questionnaires on the amount of variance explained, expressed as the relative increase in MSE when the predictor is randomly permuted across participants. The total percentage of variance explained by the Random Forest model with all Q1 to Q3 predictors is 10.91%.

Once again, we explored the effect of the different predictors on the number of days participants wore their tracker. Figures 11-15 show the marginal means (with all other factors kept constant) of the different levels of each predictor. Again, only those predictors of which the differences in marginal means implies a meaningful difference in real life (>1 day) are included: age, perceived effect on goals, user experience (valence), user experience (effect), perceived effect on goals, iPhone type, and the goal to change eating habits. User experience (valence and effect) and perceived effect on goals are continuous variables, so no levels of marginal means could be shown. Instead, we show a partial dependence plot. The user experience variables are shown normalized with a mean 0 and a SD of 1.

**Figure 10:**
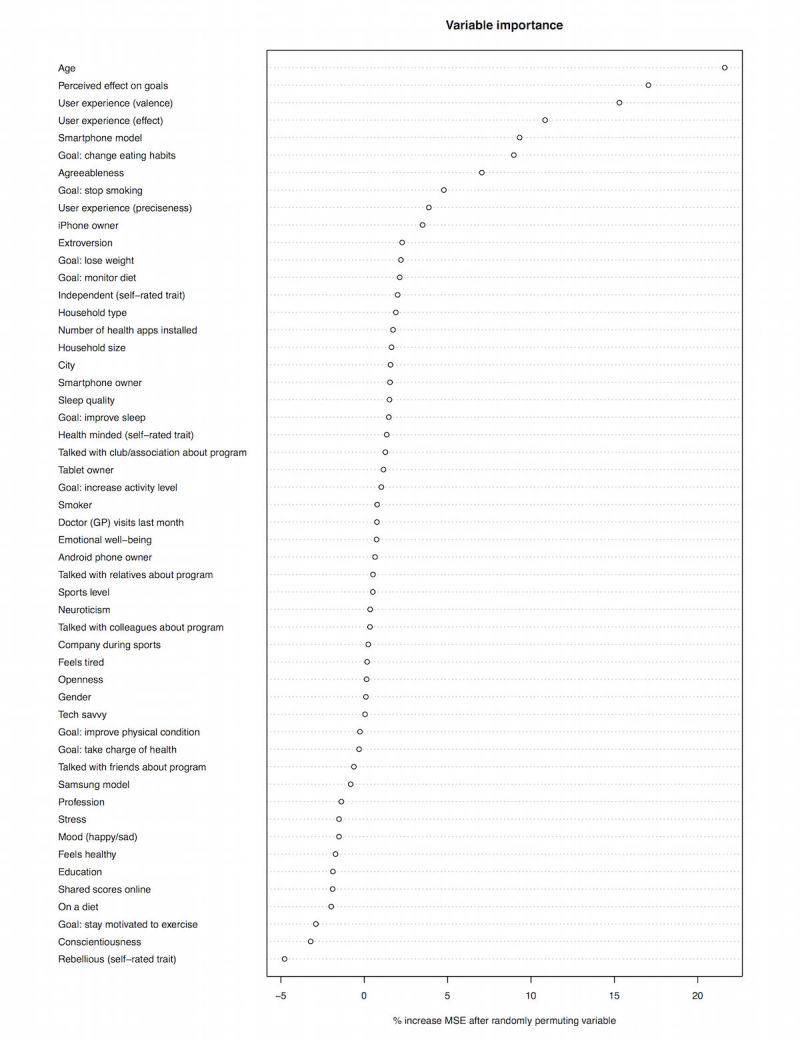
Plot of the relative importance of predictors in all questionnaires (Q1 + Q2 + Q3).

**Figure 11:**
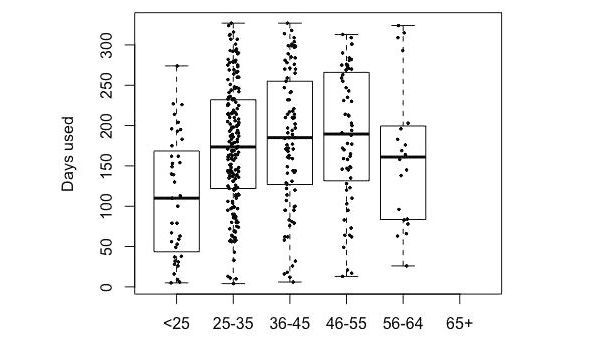
Boxplots of the distributions of the marginal means for participant age. The under-25 use the Fitbit less long than the other groups. There are no participants older than 65 in this sample.

**Figure 12:**
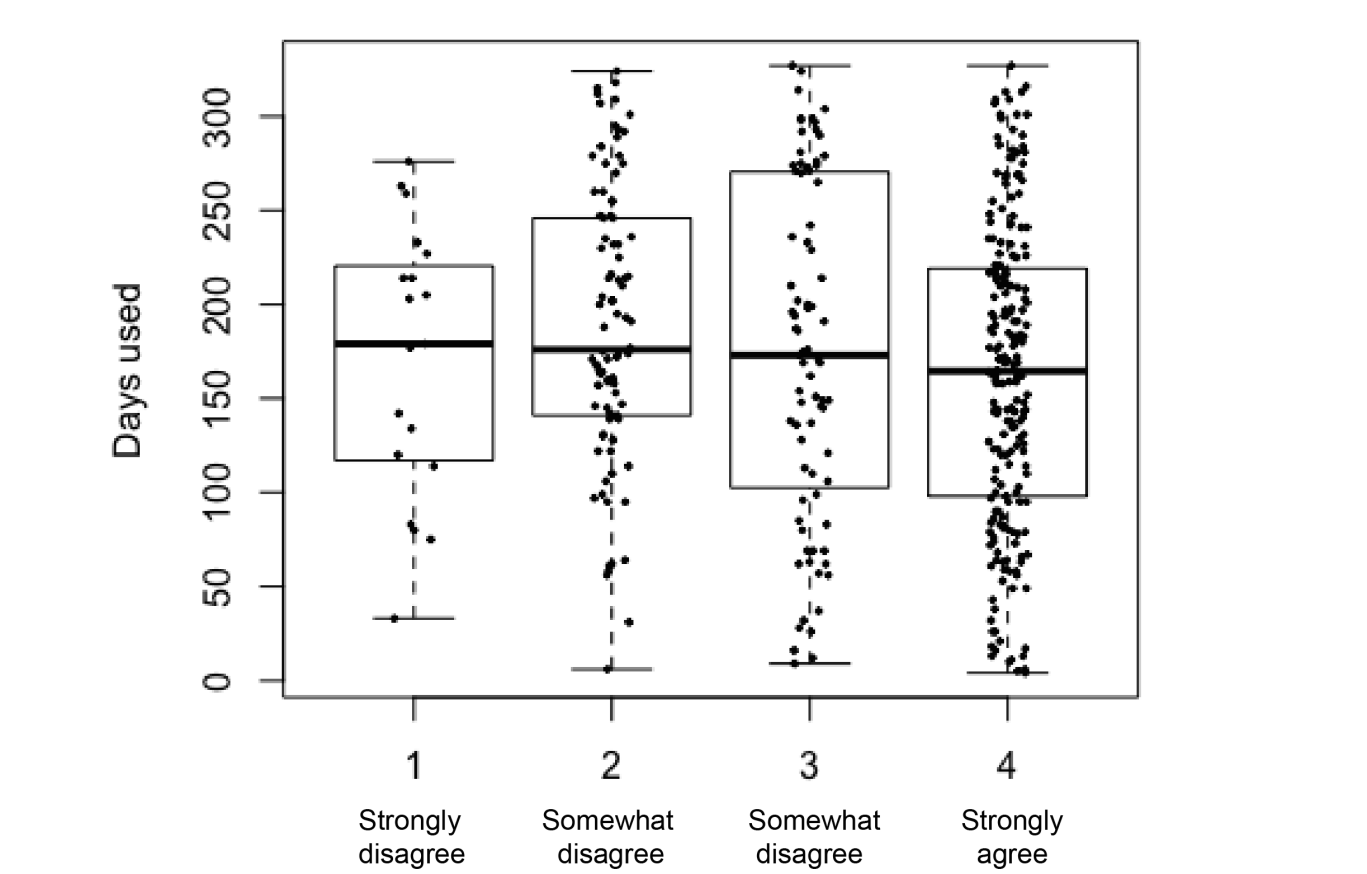
Boxplots of the distributions of having the goal to change eating habits. The stronger the goal, the less sustained use of the tracker.

**Figure 13:**
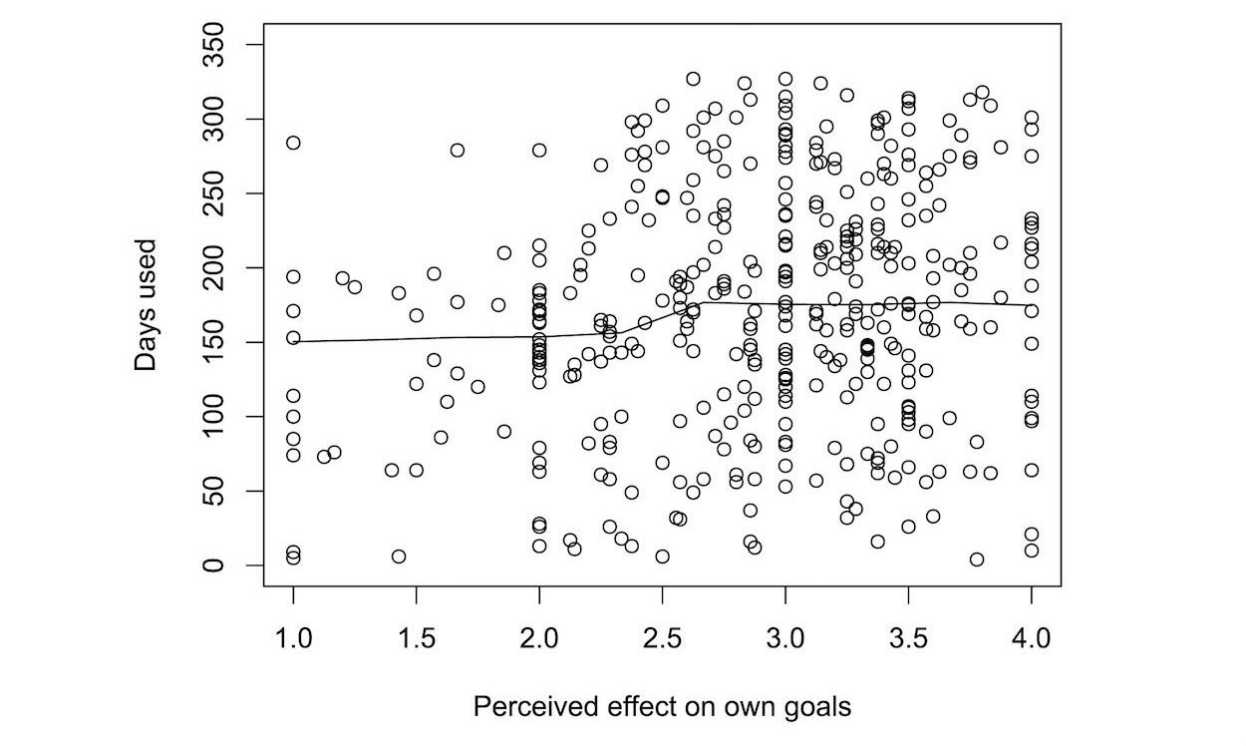
Plot of the partial dependence of sustained tracker use on the perceived effect of the tracker on goal attainment. A larger perceived effect leads to longer sustained use.

**Figure 14:**
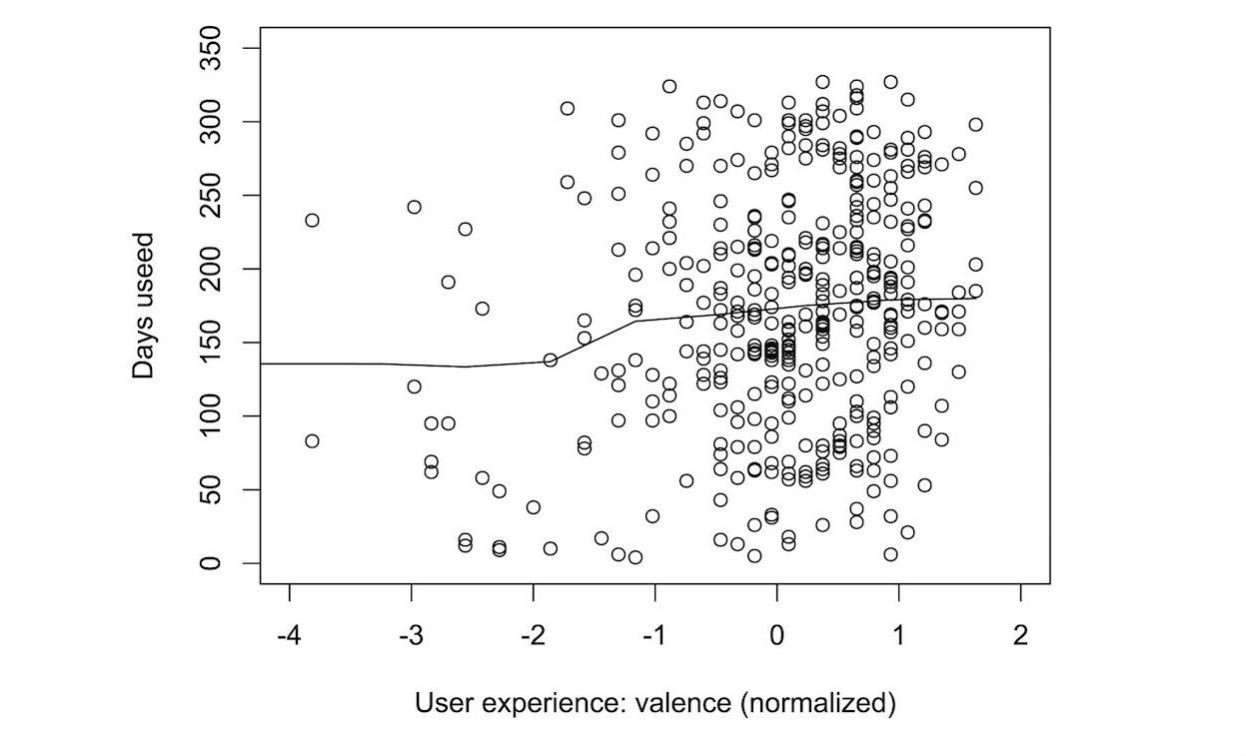
Plot of the partial dependence of sustained tracker use on user experience of the valence of the tracker. A better user experience leads to longer sustained use.

**Figure 15:**
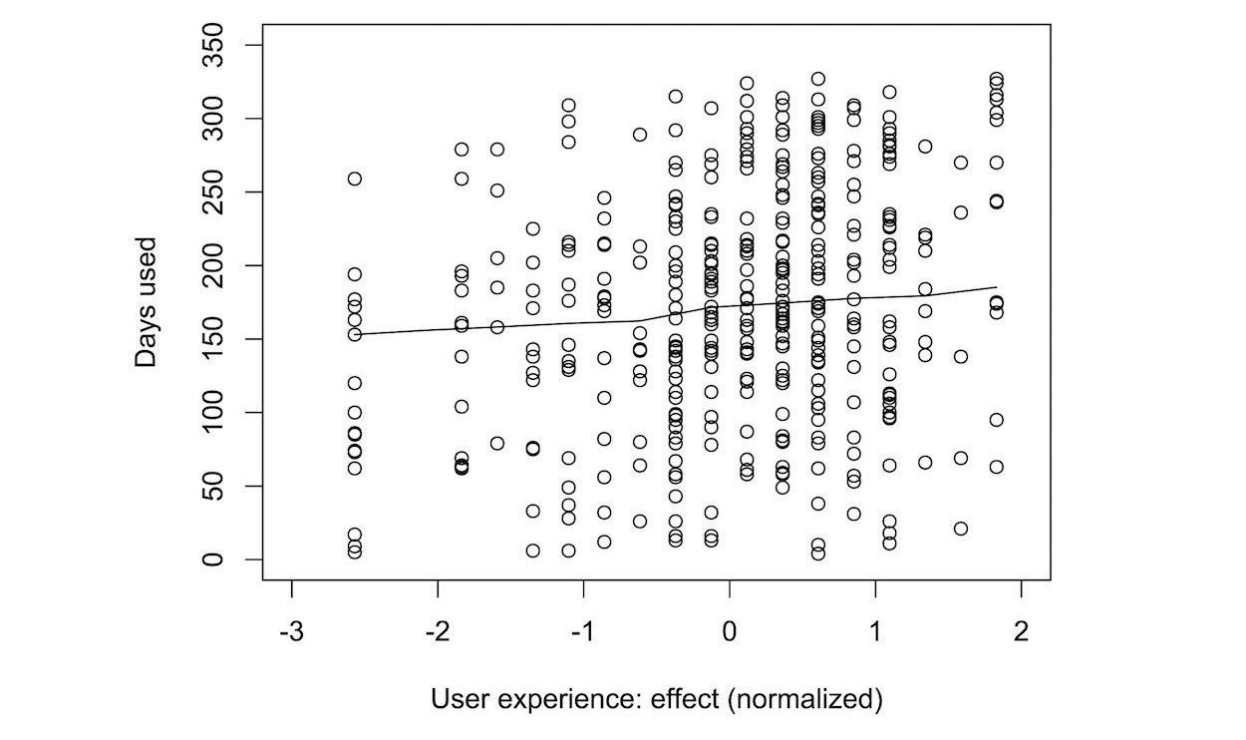
Plot of the partial dependence of sustained tracker use on user experience of the efficacy of the tracker. A better user experience leads to longer sustained use.

## Discussion

### Principal Findings

This study examined the use of an activity tracker, as well as reasons to stop using the tracker and predictors of sustained use. This study shows that of the 711 initial participants, approximately 50% still used their tracker after 6 months, and 12% continued to use their tracker even after 300 days. This rate of decay in usage confirms earlier findings [18]. This result also confirms the notion that wearable activity trackers are not subject to the rapid exponential decay of use we see in mobile phone apps, where usually 80% of users drop out in the first few days (eg, in [70]).

### Reasons to Quit Tracking

After half a year, half of the participants quit using their tracker; at the sending out of Q3, three-quarters of participants were no longer using the Fitbit. When asked for reasons for their quitting, 56.7% of those who answered the question stated some form of technical malfunctioning (including empty batteries). A further 12.8% indicated they had lost the device. This result confirms findings (eg, [21]) in which trackers were abandoned because of empty batteries, with the perceived cost of replacement too high or too cumbersome [71], but this result deviates from other findings (eg, [22,24]) in which technological failures comprise only a very small part of reasons to no longer use a device, and reasons such as sustained motivation, device aesthetics, and device accuracy seem to be of more importance. However, in studies in which reasons to quit tracking are covered, either the demand characteristics of the study (eg, [24]) or the data gathering technique (eg, [22], where advertisement data from Craigslist were used) preclude the reliable registration of technical failure as a reason to quit tracking. The results from this study may therefore serve as a first indication of the relative importance of technological reliability for the sustained use of feedback technology. Simple actions, such as changing a battery, already seem to raise insurmountable barriers for sustained use of a tracker. Newer activity trackers, fortunately, mostly do not rely on button cell batteries, which take some effort to replace, but make it possible to recharge the device, much like one would recharge a smartphone. This, however, also constitutes a barrier to sustained use. Further research into the effect of technological failures on sustained use of activity trackers, and how to help users overcome the barriers brought about by technical issues such as empty barriers, is needed to corroborate this finding and shed light on potential solution strategies.

### Relative Importance of Predictors for Self-Tracking

In the analysis of items from Q1 only (Figure 5), participant age was the only predictor that showed great impact; higher age was associated with more sustained use. A second group of predictors for sustained use were the goal to quit smoking (with not having the goal associated with longer use), iPhone type (with not having an iPhone associated with longer use), and household type (with single parents using the tracker for a shorter duration than all other groups). All other predictors were not found to have a noteworthy impact on sustained tracker use.

In the analysis of all (three) questionnaires (Figure 6), participant age was once again the strongest predictor of sustained tracker use. As in Q1, sustained use increased with age (to a point), overall (Q1 to Q3) the Fitbit was used for a shorter duration by the youngest age group (under 25). Other important predictors were user experience–related predictors (tracker valence and user experience, and perceived efficacy and helpfulness of the tracker). iPhone type, having the goal to change eating habits, and wanting to quit smoking were other relevant predictors, albeit in an opposing way: these predictors were associated with a decreased use of the self-tracking device.

The relative importance of age as a predictor of sustained tracker use (with higher age associated with longer use) is in line with previous literature (eg, [25,30,31]). More research is needed to answer the question why this is, and to determine its implications for the design of tracker-based interventions for physical activity. Is the greater efficacy for older participants problematic, or are younger participants already well-served by other possibilities to exert themselves physically? In a similar notion, is this age-effect a consequence of self-selection, in which younger people already have enough alternatives for physical activity, and it is mostly those older than 25 years that turn to tracking as viable solution? Answers to these questions also have implications for the development of tracker interventions. Do we need more age-inclusive solutions, or can we regard this type of intervention as more effective for people older than 25 years?

The importance of user experience–related predictors such as valence, perceived efficacy, and preciseness of the tracker is also in line with previous studies. User experience and ease of use [22,23], functionality or lack thereof [22], the possibility to upgrade toward a newer device (ibidem), aesthetics and form [24], and perceived fit between device and self-image [23] have all been cited as reasons to either abandon the tracker or to keep using it. This sheds light on the relative importance of technological and design-related aspects of behavior change feedback technology aimed at greater physical activity. Even though there is a substantive literature on the subject, this remains an underexposed area in current health behavior change research. Clunky intervention designs carry the risk of being rejected by their participants and, more importantly, a lack of uptake once the intervention hits the market or the app store. In health behavior change research, a lot more attention is needed for user experience, user friendliness, and the aesthetic experience.

The negative impact of goals, such as wanting to change one’s eating habits or wanting to quit smoking, seems logical in hindsight. The Fitbit tracker does not in itself contribute to the attainment of these goals, which could easily have a demoralizing effect. The fact that having an iPhone seemed to reduce the chances of sustained use could point at another covert measure of user experience. The iPhone interface for Fitbit-related feedback might possibly be more difficult to use or less functional than its Android equivalent; alternatively, iPhone users may be psychographically different from Android users on traits that lead to reduced usage of activity trackers. However, no evidence to support either hypothesis is presently available.

A surprising finding was the lack of effect of a range of predictors, which is not in line with previous literature (eg, [12,20]). *Socioeconomic status* markers such as education (eg, [25,27]) and profession [28,29], *gender* (eg, [25,27,32]), *psychological traits* (eg, [33-35]), *personal health-related factors* (eg, [36,37]), strong *motivation* (eg, in [30,31]), *strong, clear goals* (eg, in [25]), and *social interaction* (eg, in [50]) did not appear to affect tracker use. These have often been researched out of context, with predictors singled out and assessed independently. The current result could point to the fact that some predictors may not be as important as we think they are, when compared with many other possibilities. When placed in context, their role may be smaller than we assumed. A competing hypothesis, however, could be preselection; for instance, it is possible that motivation did not play a large role, because those who entered the challenge were already highly motivated. Similarly, perhaps only those already high on psychological traits such as conscientiousness took part. This preselection would limit the confidence in some of the null-results found in this study. If so, however, this preselection constitutes less of a problem as one would think. We can assume similar preselection would take place in the market place; it is reasonable to suspect that traits and states found in those who take part in this study would resemble states and traits of those people who would be interested in using a Fitbit in the first place. Unfortunately, this cannot be deducted from our research. Further research would be interesting.

The entire range of independent variables in Q1 explained 8.29% of variance; in Q1-Q3, the whole set of predictors accounted for 10.91% of explained variance. In Cohen’s [72] frequently used assessments of effect sizes for psychology, an  *R*^2 ^of .095 (Q1) to .099 (Q1-Q3) are described as a small effect or approaching a medium effect. Such an effect size is common in social and behavioral sciences, for situations where there is a lot of individual variation and many different factors may affect the dependent variable independently (see also [73]). To our best knowledge, this is the first quantitative study looking into the factors influencing the persistent use of activity trackers. Earlier studies (eg, [21,48,49,51-53]) were qualitative and small-scale studies (7 to 31 participants) and did not attempt to model activity tracker use. Thus, we have no immediate context to compare our model’s performance with.

Intuitively, we may have expected a larger effect size from such a broad range of predictors. We can discern two competing hypotheses. A first hypothesis is that sustained use is mostly predicted by random events such as empty batteries or loss, but there are many small but significant contributions from a broad range of predictors. A second hypothesis is that unmeasured third variables are responsible for the relative lack of effect. Not all relevant predictors we could identify in the literature were included in the questionnaires. First, perceived self-efficacy was not directly assessed but only through measures regarding perceived efficacy of healthier behavior change. Second, literature [49] suggests that different tracking styles exist, such as tracking physical activity to diagnose a secondary problem such as sleeping disorders or stomach problems, or “fetishized” tracking: tracking because it is cool or otherwise desirable. In this study, the tacit assumption is that all participants want to at least document and probably also change their physical activity, which might not be the case in reality. Third, different forms of intrinsic motivation, such as motivation for autonomy, mastery, and relatedness [74], might lead to different levels of adherence to activity tracking. Finally, the completion of set goals was not registered. It is plausible to assume that when people achieve their goal, their interest in tracking their progress wanes. The inclusion of these possible moderators in future research would shed light on their effect on sustained use of a tracking device. Further research could shed light on which of these possible explanations would be most feasible.

### Limiting Factors

A few limitations to this study warrant further discussion. First, our confidence in the validity of the findings is limited by the fact that of the original 929 participants, 711 gave permission to access their Fitbit data and filled out Q1; of those 711, only 575 took part in Q2 (80.9%), and 542 took part in both Q2 and Q3 (76.2%). The greater part of those participants who did not fill out Q2 or Q3 quit using their Fitbit somewhere in the period preceding that questionnaire. Even though this decline in adherence is not at all uncommon in interventions for health behavior change, and thereby no cause for alarm, their data would have increased the validity and reliability of our findings.

Similarly, 56.7% of those who provided a reason for their no longer tracking stated technical malfunctioning. Of those who did not report a reason (eg, because they did not fill out Q2 or Q3), we do not know why they no longer took part. However, the fact that at least 17.5% of all participants quit because of technical reasons still emphasizes the importance of this finding, regardless of the reasons the nonreporters could have had for quitting.

A second, and possibly greater, limitation to the validity of the findings stems from the way the study design was carried out. Questionnaire construction and data gathering were carried out by the MySantéMobile team. The quality of the questionnaires would have benefited from early involvement of social scientists with relevant experience in questionnaire construction, which would have led to a more hypothesis-based selection of questions, and more informative response scales. As it is, we think this study has enough validity to serve its purpose, that is, as an exploratory analysis of potential determinants of sustained use of physical activity trackers.

A third limitation in the study design is the fact that the psychological predictors of use such as the Big Five and user experience–related predictors were not included in Q1 but made a first appearance in Q3. This limits the applicability of findings concerning these predictors because only participants making it to Q3 (76.2% of those who filled out Q1 and gave access to their data) answered these questions. However, psychological traits are known to be stable [75], so it is reasonable to expect that no great changes in big five traits occurred. User experience-related predictors can only be measured once participants have used the product; an a-priori judgment lacks value. These, therefore, could not have been included in Q1.

Finally, the data analysis method selected has its benefits, such as robustness toward overfitting and good handling of relatively low participant populations, but Random Forest analysis also has its limitations. The result of the analysis is a ranking of the relative importance of each predictor on the use of the activity tracker. Due to its ensemble nature, results from a Random Forest can be hard to interpret (unlike a linear model). Contributions from a variable can be present in multiple ways and through nonlinear and/or (higher-order) interactions. However, through Random Forest modeling, we can establish which predictor variables are important with respect to outcome variables. These variables can be studied further to establish their effect and interactions with other variables.

### Conclusions

This study confirms earlier findings that habitual use of an activity tracker tends to decline at a slow exponential pace rather than show the rapid exponential decline shown in health app use. When they start using an activity tracker, most users in our sample continued to use it for at least half a year. Around 12% of users still use their tracker after 300 days.

This study also shows that sustained use of an activity tracker is not easy to predict. Most known predictors of sustained adherence to physical activity interventions do not seem to have an impact on sustained use in the sample observed in this study. When participants no longer use their tracker, technological failures such as empty batteries seem the predominant reason to quit.

The broad range of predictors entered in the Random Forest model in this study only led to a small proportion of explained variance. Those predictors that did have an effect on sustained use were participant age and factors related to the user experience of tracker use.

Regardless of the limitations to the findings cited above, this study shows some much-needed insight in predictors of sustained use of trackers. Furthermore, this study is one of few examples in which academia gets the chance to evaluate data from industry; the field would greatly benefit from a greater number of such collaborations, preferably with a larger role for the academic partner in setting up the study.

## Appendix

Multimedia Appendix 1 All questionnaire items with response scales, variables in which they were used, transformations, and validity evaluation.

Multimedia Appendix 2 Principal Component Analysis of items relating to User Experience.

## Abbreviations

BCT: behavior change technique
iOS: iPhone operating system
MSE: mean squared error
SES: socioeconomic status
SD: standard deviation
