# Supplementary material for: Determinants for Sustained Use of an Activity Tracker: Observational Study
Source: JMIR Mhealth Uhealth. 2017 Oct 30;5(10):e164. doi: 10.2196/mhealth.7311 (PMC5695980; doi:10.2196/mhealth.7311)
Supplement: Multimedia Appendix 1 [file mhealth_v5i10e164_app1.pdf]

Multimedia Appendix 1:
All questionnaire items with response scales, variables in which they were used, transformations, and validity evaluation

Questionnaire 1

Q1.01 City

Question: City
Response Scale
Bordeaux, Montpellier, Lille, Lyons

Used in Variable: city_1
Transformations: Transformed to numerical scale (nominal): 1 Bordeaux, 2 Montpellier, 3 Lille, 4 Lyons

Remarks:
Research has shown that single-item measures are valid when the attribute (in this case, city name) is concrete and singular and the object (in this case, place of residence) is concrete (Bergqvist & Rossiter, 2007). Thus, we can probably conclude that that these single-item self-report measures are a sufficiently valid measure for the purpose of this paper.

Q1.02 Gender

Question: Are you...? Please select one of the following: (select one)
Response Scale
Male, Female

Used in Variable: sex_1
Transformations: Transformed to numerical scale (nominal): 1 Female, 2 Male

Remarks:
Single-item measurement of concrete, singular object

Q1.03 Age

Question: What is your age group? (select one)
Response Scale
< 25, 25–35, 36–45, 46–55, 56–65, 65 >

Used in Variable: age_1
Transformations: Transformed to numerical scale (ordinal): 1 <25, 2 25-35, 3 36-45, 4 46-55, 5 56-65, 6 65+

Remarks:
Single-item measurement of concrete, singular object

Q1.04 Household size

Question: How many people live in your household?
Response Scale:
numerical

Used in Variable: household_size_1
Transformations: none

Remarks:
Single-item measurement of concrete, singular object

Q1.05 Household Structure

Question: What is your household structure? (select one)
Response Scale:
Single person, couple w/o children, single parent, couple with children, other

Used in Variable: household_type_1
Transformations: Transformed to numerical scale (nominal): 1 Single person, 2 couple w/o children, 3 single parent, 4, couple with children, 5 other

Remarks:
Single-item measurement of concrete, singular object

Q1.06 Occupation

Question: What is the head of the household's occupation? (select one)
Response Scale:
Cadre (Executive, Senior Knowledge Worker), Intermédiaire (Middle Management,), Employé (Administrative, Sales, or Service Occupations) Artisan (Farmer, craftsperson), Ouvrier (Factory worker, manual worker), Retraité (Pensioner), Sans (Unemployed)

Used in Variable: profession_1
Transformations: Transformed to numerical scale (nominal): 1 Cadre, 2 Intermédiaire, 3 Employé, 4 Artisan, 5 Ouvrier, 6 Retraité, 7 Sans

Remarks:
Single-item measurement of concrete, singular object.
The classification used in this question follows that of the French Census (cf. Niedhammer, Chastang, David, & Kelleher, 2008).

Q1.07 Owned Digital Devices

Question: Please select from the following list all the devices that you own and use on a daily basis. (select all appropriate)
Response Scale:
Personal computer Broadband Internet connection (ADSL, Box, optical fibre, etc.) iPhone, Android smartphone

Used in Variable: computer_1, broadband_1, smartphone_1, ios_1, android_1, tablet_1
Transformations: Every checkbox is transformed in a digital variable: 0 does not own/use, 1 own/use

Remarks:
Single-item measurement of concrete, singular object

Q1.07a iPhone Type

Question: Which iPhone model do you own? (depending on answer Q7)
Response Scale:
iPhone 4S, iPhone 5, other

Used in Variable: iphone_type_1
Transformations: Transformed to numerical scale (nominal): 1 Other, 2 iPhone 4S, 3 iPhone 5

Remarks:
Single-item measurement of concrete, singular object

Q1.07b Android Type

Question: Which Android smartphone model do you own? (depending on answer Q7
Response Scale:
Open

Used in Variable: samsung_type_1
Transformations: Transformed to numerical scale (nominal): 1 Other 2 Samsung Galaxy SIII

Remarks:
Single-item measurement of concrete, singular object

Q1.07c Tablet Type

Question: What model of tablet do you own? (depending on answer Q7)
Response scale:
open

Used in Variable: tablet_ 1
Transformations:

Remarks:
Single-item measurement of concrete, singular object

Q1.07d QS/Fitness apps

Question: What applications have you downloaded to your smartphone or tablet?
Please select all that apply (more answers possible)
Response scale: Select from:

Fitbit, Lose It!, My Fitnesspal, Mapmyrun, Endomondo, RunKeeper, MyKilos, DriveBit, iCardio, iRunner, Miracle, Other (field)

Used in Variable: apps_1
Transformations: apps_1 counts the total number of apps installed

Remarks:
Single-item measurement of concrete, singular object.

Q1.08 Education

Question: What is the highest degree or level of education that you have completed?
Response Scale:
Bac, Bac+2 or Bac+3, Bac+5 and further, CAP or BEP, Brevet des collèges, None (primary school only)

Used in Variable: education_1
Transformations: Transformed to numerical scale (nominal): 1 Bac, 2 Bac +2 of Bac+3, 3 Bac+5 and sup., 4 Cap or BEP, 5 Brevet des Collèges, 6 None

Remarks:
Single-item measurement of concrete, singular object
This classification follows that of the French Ministry of Education (MESRI, 2017).

Q1.09 Height

Question: How tall are you (in cm)?
Response Scale:
Numerical, in cm

Used in Variable: bmi_1 (see below)
Transformations: Height was divided by 100 to obtain height in meters. Then bmi_1 was calculated by dividing weight in kilograms by height in meters squared

Remarks: We use self-reported weight in combination with self-reported height to determine BMI, which is used in the analysis. A study among English middle-aged men and women (Spencer, Appleby, Davey, & Key, 2002) showed that self-reported BMI correlated highly with measured BMI (Spearman r >.9). Thus, we can probably conclude that self-reported BMI is a sufficiently valid measure for our purposes.

Q1.10 Weight

Question: How much do you weigh (in kg)? (If the last time you weighed yourself was a week ago, please weigh yourself again)
Response scale:
Numerical (in kilos)

Used in Variable: bmi_1
Transformations: bmi_1 was calculated by dividing weight in kilograms by height in meters squared

Remarks: See above under Q1.09

Q1.11 Smoking

Question: Do you smoke or have you smoked in the past three months?
Response Scale:
dichotomous (yes, no)

Used in Variable: smoker_1
Transformations: Transformed to numerical scale (nominal): 1 no, 2 yes

Remarks:
Single-item measurement of concrete, singular object
Smoking is a behaviour that is generally seen as undesired, and therefore susceptible to underreporting. Indeed, some reviews (i.e. Gorber et al., 2009) find that self-report of smoking is susceptible to bias, whereas other reviews (i.e. Patrick et al., 1994) show that self-reported smoking correlates highly with biochemical smoking measures. However, since bias is thought to lead to underreporting the number of cigarettes smoked per day towards the nearest round number (Klesges, Debon, & Ray, 1995) and not the denial of smoking per se, we can probably conclude that self-reported smoking is a sufficiently valid measure for our purposes.

Q1.12 Pregnancy

Question: Are you currently pregnant?
Response Scale:
dichotomous (yes, no)

Used in Variable: pregnant_1
Transformations: Transformed to numerical scale (nominal): 1 no, 2 yes

Remarks:
Single-item measurement of concrete, singular object

Q1.13 Diet

Question: Are you currently on a diet?
Response Scale
dichotomous (yes, no)

Used in Variable: diet_1
Transformations: Transformed to numerical scale (nominal): 1 no, 2 yes

Remarks:
Single-item measurement of concrete, singular object
Dietary intake is generally known as susceptible to underreporting (Subar et al., 2015). However, this generally concerns energy intake, and not the fact that a participant is currently following a diet. A single item measure of partaking in a diet is customary used in health-related studies (e.g. Geurden et al., 2014; Kelly et al., 2016; Li et al., 2016; and others). We therefore conclude that self-reported diet is a sufficiently valid measure for our purposes.

Q1.14 GP visits

Question: In the past month, how many times did you see your general practitioner?
Response Scale
Open

Used in Variable: visits_1
Transformations: None

Remarks:
A study among 303 primary care attenders in the UK (Patel et al., 2005) showed that there is good agreement between GP records and self-reported GP attendance. Thus, we can probably conclude that a self-report measure of GP visits is a sufficiently valid measure for our purposes.

Q1.15 Sports

Question: When it comes to playing sports, what statement is most applicable?
Response Scale
You play a sport occasionally (less than once a week)
You play a sport regularly (at least once a week)
You play a sport intensively (at least twice a per week)
You play a sport at a high level or professionally

Used in Variable: sport_level_1
Transformations: Transformed to numerical scale (nominal): 1 Occasionally, 2 Regularly, 3 Intensively, 4 High level

Remarks:
Single-item measurement of concrete, singular object
A review of existing literature (Sallis & Saelens, 2000) suggests that self-report of vigorous physical activity (e.g. participating in sports activities) are relatively valid when compared to self-report measures of physical activity in general. Thus, we can probably conclude that a self-report measure of sports activity is a sufficiently valid measure for the purpose of this paper.

Q1.16 Sports in Company

Question: When you were doing sports, were you mostly...
Response Scale:
alone, with a family member with a friend, with a colleague, in a team

Used in Variable: sport_company_1
Transformations: Transformed to numerical scale (nominal): 1 alone, 2 with a family member, 3 with a friend, 4 with a colleague, 5 in a team

Remarks:
Single-item measurement of concrete, singular object
See above under Q1.15 Sports

Q1.17 Sleep quality

Question: How would you rate the quality of your sleep right now?
Response Scale:
1 – 10 (1 very poor t/m 10 excellent)

Used in Variable: sleep_quality_1
Transformations: None

Remarks:
The gold standard of sleep quality assessment is the Pittsburgh Sleep Quality Index (PSQI, Buysse et al., 1989). The single-item measurement in the questionnaire does not offer the same validity as the PSQI, which consists of a range of items assessed in a longer period of time. However, single-item measurements of sleep quality have also been found useful and valid (e.g. Jenkins et al., 1988; Cappelleri et al., 2009). Thus, we can probably conclude that a single item self-report measure of sleep quality is a sufficiently valid measure for the purpose of this paper.

Q1.18 Emotional well-being

Question: How would you rate your emotional well-being right now?
Response Scale
1 – 10 (1 very poor t/m 10 excellent)

Used in Variable: mood_1
Transformations: None

Remarks:
This range of questions (Q1.18 – Q1.24) all assess emotional wellbeing. Single-item assessments of emotional wellbeing are often used in large scale surveys (see Cooke, Melchert, & Connor, 2016 for an overview) and have been shown to perform quite well compared to multiple-items scales (e.g., Jovanović, 2016). We can therefore probably conclude that these single-item self-report measures are a sufficiently valid measure for the purpose of this paper.

Q1.19 Fatigue

Question: Last month, did you feel tired during the day?
Response Scale:
strongly disagree, somewhat disagree, somewhat agree, strongly agree

Used in Variable: tired_1
Transformations: Transformed to numerical scale (ordinal): 1 Strongly Disagree 2 Somewhat Disagree 3 Somewhat Agree 4 Strongly Agree

Remarks:
See above, under Q1.18

Q1.20 General health

Question: Last month, did you feel healthy?
Response Scale:
strongly disagree, somewhat disagree, somewhat agree, strongly agree

Used in Variable: well_1
Transformations: Transformed to numerical scale (ordinal): 1 Strongly Disagree 2 Somewhat Disagree 3 Somewhat Agree 4 Strongly Agree

Remarks:
See above, under Q1.18

Q1.21 Sadness

Question: Last month, did you feel sad?
Response Scale:
strongly disagree, somewhat disagree, somewhat agree, strongly agree

Used in Variable: affect_1
Transformations: Transformed to numerical scale (ordinal): 1 Strongly Disagree 2 Somewhat Disagree 3 Somewhat Agree 4 Strongly Agree
Variable affect_1 was calculated by subtracting Sadness score from happiness score (see below)
Variable affect_123, a measure of emotional wellbeing derived from items about happiness and sadness in all three questionnaires (Q1.21, Q1.22, Q2.01, Q2.02, Q3.01 and Q3.02) was calculated by subtracting the sum of all three sadness items (Q1.21 + Q2.02 + Q3.02) divided by three from the sum of all three happiness items (Q1.22 + Q2.01 + Q3.01) divided by three.

Remarks:
See above, under Q1.18

Q1.22 Happiness

Question: Last month, did you feel happy?
Response Scale:
strongly disagree, somewhat disagree, somewhat agree, strongly agree

Used in Variable: affect_1
Transformations: Transformed to numerical scale (ordinal): 1 Strongly Disagree 2 Somewhat Disagree 3 Somewhat Agree 4 Strongly Agree
Variable affect_1 was calculated by subtracting Sadness score from happiness score (see above)
Variable affect_123, a measure of emotional wellbeing derived from items about happiness and sadness in all three questionnaires (Q1.21, Q1.22, Q2.01, Q2.02, Q3.01 and Q3.02) was calculated by subtracting the sum of all three sadness items (Q1.21 + Q2.02 + Q3.02) divided by three from the sum of all three happiness items (Q1.22 + Q2.01 + Q3.01) divided by three.

Remarks:
See above, under Q1.18

Q1.23 Stress

Question: Last month, did you feel stressed?
Response Scale:
strongly disagree, somewhat disagree, somewhat agree, strongly agree

Used in Variable: stress_1
Transformations: Transformed to numerical scale (ordinal): 1 Strongly Disagree 2 Somewhat Disagree 3 Somewhat Agree 4 Strongly Agree
Variable Stressed_1 was calculated by subtracting the result of item Q1.24 (Calmness) from the result of item Q1.23 (Stress).

Remarks:
See above, under Q1.18

Q1.24 Calmness

Question: Last month, did you feel calm?
Response Scale:
strongly disagree, somewhat disagree, somewhat agree, strongly agree

Used in Variable: calm_1
Transformations: Transformed to numerical scale (ordinal): 1 Strongly Disagree 2 Somewhat Disagree 3 Somewhat Agree 4 Strongly Agree
Variable Stressed_1 was calculated by subtracting the result of item Q1.24 (Calmness) from the result of item Q1.23 (Stress).

Remarks:
See above, under Q1.18

Q1.25 Goal: increasing activity

Question: Do you have the goal to increase your activity?
Response Scale:
strongly disagree, somewhat disagree, somewhat agree, strongly agree

Used in Variable: goal_active_1
Transformations: Transformed to numerical scale (ordinal): 1 Strongly Disagree 2 Somewhat Disagree 3 Somewhat Agree 4 Strongly Agree

Remarks:
Fitness goals (Q1.25-Q1.33) were assessed using 9 items, each assessing a different goal: respondents were asked to indicate on a 4-pt Likert scale (1= strongly disagree, 4 =strongly agree) whether they had the goal to improve sleep / quit smoking / observe nutrition / change nutrition / increase the motivation for physical activity / observe health / increase activity / lose weight. Each item was used as a single item measure of the specific goal that was assessed. Research has shown that single-item measures are valid when the attribute (in this case, agreement) is concrete and singular and the object (in this case, a fitness goal) is concrete (Bergqvist & Rossiter, 2007). Thus, we can probably conclude that that these single-item self-report measures are a sufficiently valid measure for the purpose of this paper.

Q1.26 Goal: improving sleep

Question: Do you have the goal to improve your sleep?
Response Scale:
strongly disagree, somewhat disagree, somewhat agree, strongly agree

Used in Variable: goal_sleep_1
Transformations: Transformed to numerical scale (ordinal): 1 Strongly Disagree 2 Somewhat Disagree 3 Somewhat Agree 4 Strongly Agree

Remarks:
See above, under Q1.25

Q1.27 Goal: quit smoking

Question: Do you have the goal to quit smoking?
Response Scale:
strongly disagree, somewhat disagree, somewhat agree, strongly agree

Used in Variable: goal_smoking_1
Transformations: Transformed to numerical scale (ordinal): 1 Strongly Disagree 2 Somewhat Disagree 3 Somewhat Agree 4 Strongly Agree

Remarks:
See above, under Q1.25

Q1.28 Goal: observing nutrition

Question: Do you have the goal to observe your nutrition?
Response Scale:
strongly disagree, somewhat disagree, somewhat agree, strongly agree

Used in Variable: goal_food_watch_1
Transformations: Transformed to numerical scale (ordinal): 1 Strongly Disagree 2 Somewhat Disagree 3 Somewhat Agree 4 Strongly Agree

Remarks:
See above, under Q1.25

Q1.29 Goal: changing nutrition

Question: Do you have the goal to change your nutrition?
Response Scale:
strongly disagree, somewhat disagree, somewhat agree, strongly agree

Used in Variable: goal_eating_habits_1

Transformations: Transformed to numerical scale (ordinal): 1 Strongly Disagree 2 Somewhat Disagree 3 Somewhat Agree 4 Strongly Agree

Remarks:
See above, under Q1.25

Q1.30 Goal: increasing motivation for physical activity

Question: Do you have the goal to increase your motivation for physical activity?
Response Scale:
strongly disagree, somewhat disagree, somewhat agree, strongly agree

Used in Variable: goal_motivation_1
Transformations: Transformed to numerical scale (ordinal): 1 Strongly Disagree 2 Somewhat Disagree 3 Somewhat Agree 4 Strongly Agree

Remarks:
See above, under Q1.25

Q1.31 Goal: observing health

Question: Do you have the goal to observe your health?
Response Scale:
strongly disagree, somewhat disagree, somewhat agree, strongly agree

Used in Variable: goal_health_1
Transformations: Transformed to numerical scale (ordinal): 1 Strongly Disagree 2 Somewhat Disagree 3 Somewhat Agree 4 Strongly Agree

Remarks:
See above, under Q1.25

Q1.32 Goal: weight loss

Question: Do you have the goal to lose weight?
Response Scale:
strongly disagree, somewhat disagree, somewhat agree, strongly agree

Used in Variable: goal_weight_1
Transformations: Transformed to numerical scale (ordinal): 1 Strongly Disagree 2 Somewhat Disagree 3 Somewhat Agree 4 Strongly Agree

Remarks:
See above, under Q1.25

Q1.33 Goal: improving physical condition

Question: Do you have the goal to improve your physical condition?

Response Scale:
strongly disagree, somewhat disagree, somewhat agree, strongly agree

Used in Variable: goal_shape_1
Transformations: Transformed to numerical scale (ordinal): 1 Strongly Disagree 2 Somewhat Disagree 3 Somewhat Agree 4 Strongly Agree

Remarks:
See above, under Q1.25

Not Used:
QN1.01 Medication

Question: In the past month, have you had to take medication every day?
Response Scale:
Yes, No

Remarks:
The wording of the question leads to confusion. What constitutes medication? What about medication which does not have to be taken every day, but still leads to changes in physical activity?

QN1.02 General activity

Question: Are you an active person?
Response Scale:
Yes, No

Remarks:
The dichotomous response scale does not leave room for the nuance many people would like to add to their answer. Furthermore, social desirability makes it unlikely that people entering a study using activity trackers would answer this question with a plain 'no'.

Questionnaire 2

Q2.01 Happiness

Question: Last month, did you feel happy?
Response Scale:
strongly disagree, somewhat disagree, somewhat agree, strongly agree

Used in Variable: affect_2
Transformations: Transformed to numerical scale (ordinal): 1 Strongly Disagree 2 Somewhat Disagree 3 Somewhat Agree 4 Strongly Agree
Variable affect_123, a measure of emotional wellbeing derived from items about happiness and sadness in all three questionnaires (Q1.21, Q1.22, Q2.01, Q2.02, Q3.01 and Q3.02) was calculated by subtracting the sum of all three sadness items (Q1.21 + Q2.02 + Q3.02) divided by three from the sum of all three happiness items (Q1.22 + Q2.01 + Q3.01) divided by three.

Remarks:
This question and the following (Q2.01 – Q2.04) all assess emotional wellbeing. Single-item assessments of emotional wellbeing are often used in large scale surveys (see Cooke, Melchert, & Connor, 2016 for an overview) and have been shown to perform quite well compared to multiple-items scales (e.g., Jovanović, 2016). We can therefore probably conclude that these single-item self-report measures are a sufficiently valid measure for the purpose of this paper.

Q2.02 Sadness

Question: Last month, did you feel sad?
Response Scale:
strongly disagree, somewhat disagree, somewhat agree, strongly agree

Used in Variable: affect_2
Transformations: Transformed to numerical scale (ordinal): 1 Strongly Disagree 2 Somewhat Disagree 3 Somewhat Agree 4 Strongly Agree
Variable affect_123, a measure of emotional wellbeing derived from items about happiness and sadness in all three questionnaires (Q1.21, Q1.22, Q2.01, Q2.02, Q3.01 and Q3.02) was calculated by subtracting the sum of all three sadness items (Q1.21 + Q2.02 + Q3.02) divided by three from the sum of all three happiness items (Q1.22 + Q2.01 + Q3.01) divided by three.

Remarks:
See above, under Q2.01

Q2.03 Stress

Question: Last month, did you feel stressed?
Response Scale:
strongly disagree, somewhat disagree, somewhat agree, strongly agree

Used in Variable: stress_2
Transformations: Transformed to numerical scale (ordinal): 1 Strongly Disagree 2 Somewhat Disagree 3 Somewhat Agree 4 Strongly Agree

Variable Stressed_123 was calculated by subtracting the sum of items Q1.24, Q2.04 and Q3.04 (Calmness) divided by 3 from the sum of items Q1.23, Q2.03 and Q3.03 (Stress) divided by 3.

Remarks:
See above, under Q2.01

Q2.04 Calmness

Question: Last month, did you feel calm?
Response Scale:
strongly disagree, somewhat disagree, somewhat agree, strongly agree

Used in Variable: calm_2
Transformations: Transformed to numerical scale (ordinal): 1 Strongly Disagree 2 Somewhat Disagree 3 Somewhat Agree 4 Strongly Agree
Variable Stressed_123 was calculated by subtracting the sum of items Q1.24, Q2.04 and Q3.04 (Calmness) divided by 3 from the sum of items Q1.23, Q2.03 and Q3.03 (Stress) divided by 3.

Remarks:
See above, under Q2.01

Q2.05 Online Social Sharing

Question: Did you share your scores on... Select all that apply
Response Scale:
Your social media profile, Your website Twitter, A blog, A forum, A messaging service

Used in Variable: share_online_23
Transformations: if participant selected one or more of these items OR if participant selected one or more of these items in questionnaire 3 (Q3.05), share_online_23 was set to 1; otherwise, share_online_23 was set to 0.

Remarks:
Single-item measurement of concrete, singular object

Q2.06 Offline Social Sharing

Question: Whom did you tell about the My Santé Mobile programme? Select all that apply
Response Scale:
A family member, A friend, A colleague, Members of a group (club, association, etc.)

Used in variables: talked_relative_2 talked_friends_2 talked_colleagues_2 talked_club_2
Transformations: if participant selected 'a family member' here or in Q3 (Q3.07), talked_relative_23 was set to 1, otherwise 0
if participant selected 'a family member' here or in Q3 (Q3.07), talked_friends_23 was set to 1, otherwise 0
if participant selected 'a friend' here or in Q3 (Q3.07), talked_club_23 was set to 1, otherwise 0
if participant selected 'a colleague' here or in Q3 (Q3.07), talked_colleagues_23 was set to 1, otherwise 0

if participant selected 'members of a group' here or in Q3 (Q3.07), talked_club_23 was set to 1, otherwise 0

Remarks:
Single-item measurement of concrete, singular object

Q2.07 Useful

Question: What do you think of the Fitbit? For each of the following statements, indicate if you strongly agree, somewhat agree, somewhat disagree, or strongly disagree. You think the Fitbit is USEFUL
Response Scale:
strongly disagree, somewhat disagree, somewhat agree, strongly agree

Used in variable: feat_practical_2
Transformations: Transformed to numerical scale (ordinal): 1 Strongly Disagree 2 Somewhat Disagree 3 Somewhat Agree 4 Strongly Agree
Scores on items Q2.07 – Q2.18 from Questionnaire 2, and items Q3.23 – Q3.40 from Questionnaire 3 were used in a factor analysis, which informed the construction of three scales:
1) Valence (UX_valence), formed by the sum of the items usefulness / practicality (Q2.07), enjoyableness (Q2.08), modernity (Q2.10), fun (Q2.11), credibility (Q3.34), ease of use (Q2.13), level of answering to needs (Q2.15), beauty (Q2.18), and robustness (Q2.19), divided by 9, minus the sum of the items intrusiveness (Q2.09), inconvenience (Q2.14, and cumbersomeness / nuisance (Q2.16), divided by 3.
2) Preciseness (UX_precise), formed by the sum of the items exactness (Q3.31), level of detail (Q3.32), clarity (Q3.33), and reliability (Q2.12), divided by 4, and
3) perceived efficacy (UX_effect), formed by the sum of the items perceived effect on activity increase (Q3.37), perceived effect on health changes (Q3.38), and wellbeing (Q3.39), divided by three.
The results of the factor analysis (PCA) can be found in table 1 (items Q2.07 – Q2.18) and 2 (items Q3.23 – Q3.40) at the end of this multimedia appendix.

Remarks
The three UX-related factors Valence, Preciseness, and Perceived Efficacy match similar factors in user experience research (e.g. Hassenzahl, 2003). Therefore we may conclude that the results of the factor analysis are valid enough for the purpose of this study.
The results of the factor analysis (PCA) can be found in table 1 (items Q2.07 – Q2.18) and 2 (items Q3.23 – Q3.40) at the end of this multimedia appendix.

Q2.08 Enjoyable

Question: What do you think of the Fitbit? For each of the following statements, indicate if you strongly agree, somewhat agree, somewhat disagree, or strongly disagree. You think the Fitbit is ENJOYABLE
Response Scale:
strongly disagree, somewhat disagree, somewhat agree, strongly agree

Used in variable: feat_nice_2
Transformations: Transformed to numerical scale (ordinal): 1 Strongly Disagree 2 Somewhat Disagree 3 Somewhat Agree 4 Strongly Agree

Remarks
See above under Q2.07

Q2.09 Intrusive

Question: What do you think of the Fitbit? For each of the following statements, indicate if you strongly agree, somewhat agree, somewhat disagree, or strongly disagree. You think the Fitbit is INTRUSIVE
Response Scale:
strongly disagree, somewhat disagree, somewhat agree, strongly agree

Used in variable: feat_intrusive_2
Transformations: Transformed to numerical scale (ordinal): 1 Strongly Disagree 2 Somewhat Disagree 3 Somewhat Agree 4 Strongly Agree

Remarks
See above under Q2.07

Q2.10 Modern

Question: What do you think of the Fitbit? For each of the following statements, indicate if you strongly agree, somewhat agree, somewhat disagree, or strongly disagree. You think the Fitbit is MODERN
Response Scale:
strongly disagree, somewhat disagree, somewhat agree, strongly agree

Used in variable: feat_modern_2
Transformations: Transformed to numerical scale (ordinal): 1 Strongly Disagree 2 Somewhat Disagree 3 Somewhat Agree 4 Strongly Agree

Remarks
See above under Q2.07

Q2.11 Fun

Question: What do you think of the Fitbit? For each of the following statements, indicate if you strongly agree, somewhat agree, somewhat disagree, or strongly disagree. You think the Fitbit is FUN
Response Scale:
strongly disagree, somewhat disagree, somewhat agree, strongly agree

Used in variable: feat_amusing_2
Transformations: Transformed to numerical scale (ordinal): 1 Strongly Disagree 2 Somewhat Disagree 3 Somewhat Agree 4 Strongly Agree

Remarks
See above under Q2.07

Q2.12 Reliable

Question: What do you think of the Fitbit? For each of the following statements, indicate if you strongly agree, somewhat agree, somewhat disagree, or strongly disagree. You think the Fitbit is RELIABLE
Response Scale:
strongly disagree, somewhat disagree, somewhat agree, strongly agree

Used in variable: feat_credible_2
Transformations: Transformed to numerical scale (ordinal): 1 Strongly Disagree 2 Somewhat Disagree 3 Somewhat Agree 4 Strongly Agree

Remarks
See above under Q2.07

Q 2.13 Simple to use

Question: What do you think of the Fitbit? For each of the following statements, indicate if you strongly agree, somewhat agree, somewhat disagree, or strongly disagree. You think the Fitbit is SIMPLE TO USE
Response Scale:
strongly disagree, somewhat disagree, somewhat agree, strongly agree

Used in variable: feat_easy_2
Transformations: Transformed to numerical scale (ordinal): 1 Strongly Disagree 2 Somewhat Disagree 3 Somewhat Agree 4 Strongly Agree
See above under Q2.07

Remarks
See above under Q2.07

Q2.14 Inconvenient

Question: What do you think of the Fitbit? For each of the following statements, indicate if you strongly agree, somewhat agree, somewhat disagree, or strongly disagree. You think the Fitbit is INCONVENIENT
Response Scale:
strongly disagree, somewhat disagree, somewhat agree, strongly agree

Used in variable: feat_embarassing_2
Transformations: Transformed to numerical scale (ordinal): 1 Strongly Disagree 2 Somewhat Disagree 3 Somewhat Agree 4 Strongly Agree

Remarks
See above under Q2.07

Q2.15 Meets your needs

Question: What do you think of the Fitbit? For each of the following statements, indicate if you strongly agree, somewhat agree, somewhat disagree, or strongly disagree. You think the Fitbit is MEETING YOUR NEEDS
Response Scale:
strongly disagree, somewhat disagree, somewhat agree, strongly agree

Used in variable: feat_needs_2
Transformations: Transformed to numerical scale (ordinal): 1 Strongly Disagree 2 Somewhat Disagree 3 Somewhat Agree 4 Strongly Agree

Remarks
See above under Q2.07

Q2.16 Cumbersome

]Question: What do you think of the Fitbit? For each of the following statements, indicate if you strongly agree, somewhat agree, somewhat disagree, or strongly disagree. You think the Fitbit is CUMBERSOME
Response Scale:
strongly disagree, somewhat disagree, somewhat agree, strongly agree

Used in variable: feat_nuisance_2
Transformations: Transformed to numerical scale (ordinal): 1 Strongly Disagree 2 Somewhat Disagree 3 Somewhat Agree 4 Strongly Agree

Remarks
See above under Q2.07

Q2.17 Beautifuly designed

Question: What do you think of the Fitbit? For each of the following statements, indicate if you strongly agree, somewhat agree, somewhat disagree, or strongly disagree. You think the Fitbit is BEAUTIFULLY DESIGNED
Response Scale:
strongly disagree, somewhat disagree, somewhat agree, strongly agree

Used in variable: feat_beautiful_2
Transformations: Transformed to numerical scale (ordinal): 1 Strongly Disagree 2 Somewhat Disagree 3 Somewhat Agree 4 Strongly Agree

Remarks
See above under Q2.07

Q2.18 Robustness / Sturdiness

Question: What do you think of the Fitbit? For each of the following statements, indicate if you strongly agree, somewhat agree, somewhat disagree, or strongly disagree. You think the Fitbit is STURDY
Response Scale:

strongly disagree, somewhat disagree, somewhat agree, strongly agree

Used in variable: feat_robust_2
Transformations: Transformed to numerical scale (ordinal): 1 Strongly Disagree 2 Somewhat Disagree 3 Somewhat Agree 4 Strongly Agree

Remarks
See above under Q2.07

Questionnaire 3

Q3.01 Happiness

Question: Last month, did you feel happy?
Response Scale:
strongly disagree, somewhat disagree, somewhat agree, strongly agree

Used in Variable: happy_3
Transformations: Transformed to numerical scale (ordinal): 1 Strongly Disagree 2 Somewhat Disagree 3 Somewhat Agree 4 Strongly Agree
Variable affect_123, a measure of emotional wellbeing derived from items about happiness and sadness in all three questionnaires (Q1.21, Q1.22, Q2.01, Q2.02, Q3.01 and Q3.02) was calculated by subtracting the sum of all three sadness items (Q1.22 + Q2.02 + Q3.02) divided by three from the sum of all three happiness items (Q1.21 + Q2.01 + Q3.01) divided by three.

Remarks:
This question and the following (Q3.01 – Q3.02) assess emotional wellbeing. Single-item assessments of emotional wellbeing are often used in large scale surveys (see Cooke, Melchert, & Connor, 2016 for an overview) and have been shown to perform quite well compared to multiple-items scales (e.g., Jovanović, 2016). We can therefore probably conclude that these single-item self-report measures are a sufficiently valid measure for the purpose of this paper.

Q3.02 Sadness

Question: Last month, did you feel sad?
Response Scale:
strongly disagree, somewhat disagree, somewhat agree, strongly agree

Used in Variable: sad_3
Transformations:
Transformations: Transformed to numerical scale (ordinal): 1 Strongly Disagree 2 Somewhat Disagree 3 Somewhat Agree 4 Strongly Agree
Variable affect_123, a measure of emotional wellbeing derived from items about happiness and sadness in all three questionnaires (Q1.21, Q1.22, Q2.01, Q2.02, Q3.01 and Q3.02) was calculated by subtracting the sum of all three sadness items (Q1.22 + Q2.02 + Q3.02) divided by three from the sum of all three happiness items (Q1.21 + Q2.01 + Q3.01) divided by three.

Remarks:
See above, under Q3.01

Q3.03 Stress

Question: Last month, did you feel stressed?
Response Scale:
strongly disagree, somewhat disagree, somewhat agree, strongly agree

Used in Variable: stress_3
Transformations:

Variable Stressed_123 was calculated by subtracting the sum of items Q1.24, Q2.04 and Q3.04 (Calmness) divided by 3 from the sum of items Q1.23, Q2.03 and Q3.03 (Stress) divided by 3.

Remarks:
See above, under Q3.01

Q3.04 Calmness

Question: Last month, did you feel calm?
Response Scale:
strongly disagree, somewhat disagree, somewhat agree, strongly agree

Used in Variable: calm_3
Transformations:
Variable Stressed_123 was calculated by subtracting the sum of items Q1.24, Q2.04 and Q3.04 (Calmness) divided by 3 from the sum of items Q1.23, Q2.03 and Q3.03 (Stress) divided by 3.

Remarks:
See above, under Q3.01

Q3.05 Online Social Sharing

Question: Did you share your scores on... Select all that apply
Response Scale:
Your social media profile, Your website Twitter, A blog, A forum, A messaging service

Used in Variable: share_online_23
online social sharing
Transformations: if participant selected one or more of these items OR if participant selected one or more of these items in questionnaire 2 (Q2.05), share_online_23 was set to 1; otherwise, share_online_23 was set to 0.

Remarks:
Single-item measurement of concrete, singular object

Q3.06 Offline Social Sharing

Question: Whom did you tell about the My Santé Mobile programme? Select all that apply
Response Scale:
A family member, A friend, A colleague, Members of a group (club, association, etc.)

Used in variables: talked_relative_3, talked_friends_3, talked_colleagues_3, talked_club_3
Transformations: if participant selected 'a family member' here or in Q2 (Q2.06), talked_relative_23 was set to 1, otherwise 0
if participant selected 'a family member' here or in Q2 (Q2.06), talked_friends_23 was set to 1, otherwise 0
if participant selected 'a friend' here or in Q2 (Q2.06), talked_club_23 was set to 1, otherwise 0
if participant selected 'a colleague' here or in Q2 (Q2.06), talked_colleagues_23 was set to 1, otherwise 0

if participant selected 'members of a group' here or in Q2 (Q2.06), talked_club_23 was set to 1, otherwise 0

Remarks:
Single-item measurement of concrete, singular object

Q3.07 Neuroticism, negatively phrased

Question: Here is a list of character traits that which may or may not describe you. Please indicate for each trait whether you think you possess this trait:
Anxious, Easily Troubled
Response Scale:
7-point scale ranging from '1 - not at all agree' to '7 - totally agree'

Used in variable: neurotic_neg_3
Transformations: The variable neurotic_3 was calculated by subtracting the negatively phrased item on neuroticism (variable neurotic_neg_3, Q.3.07) from the positively phrased item on neuroticism (variable neurotic_pos_3, Q3.16).

Remarks:
These questions (Q3.07 – Q3.17) assess personality using Ten-Item Personality Inventory, which is generally considered a valid instrument to measure these constructs (Storme, Tavani, & Myszkowski, 2016).

Q3.08 Extraversion, negatively phrased

Question: Here is a list of character traits that which may or may not describe you. Please indicate for each trait whether you think you possess this trait:
Reserved, Quiet
Response Scale:
7-point scale ranging from '1 - not at all agree' to '7 - totally agree'

Used in variable: extravert_neg_3
Transformations: The variable extravert_3 was calculated by subtracting the negatively phrased item on extraversion (extravert_neg_3, Q.3.08) from the positively phrased item on extraversion (extravert_pos_3, Q3.09).

Remarks:
See above, under Q3.07

Q3.09 Extraversion, positively phrased

Question: Here is a list of character traits that which may or may not describe you. Please indicate for each trait whether you think you possess this trait:
Extravert, Enthousiastic
Response Scale:
7-point scale ranging from '1 - not at all agree' to '7 - totally agree'

Used in variable: extravert_pos_3

Transformations: The variable extravert_3 was calculated by subtracting the negatively phrased item on extraversion (extravert_neg_3, Q.3.08) from the positively phrased item on extraversion (extravert_pos_3, Q3.09).

Remarks:
See above under Q3.07.

Q3.10 Agreeableness, negatively phrased

Question: Here is a list of character traits that which may or may not describe you. Please indicate for each trait whether you think you possess this trait:
Critical, aggressive
Response Scale:
7-point scale ranging from '1 - not at all agree' to '7 - totally agree'

Used in variable: agreeable_neg_3
Transformations: The variable agreeable_3 was calculated by subtracting the negatively phrased item on extraversion (agreeable_neg_3, Q.3.10) from the positively phrased item on extraversion (agreeable_pos_3, Q3.14).

Remarks:
See above, under Q3.07

Q3.12 Conscientiousness, positively phrased

Question: Here is a list of character traits that which may or may not describe you. Please indicate for each trait whether you think you possess this trait:
Conscientious, self-disciplined
Response Scale:
7-point scale ranging from '1 - not at all agree' to '7 - totally agree'

Used in variable: consc_pos_3
Transformations: The variable consc_3 was calculated by subtracting the negatively phrased item on conscientiousness (consc_neg_3, Q.3.15) from the positively phrased item on conscientiousness (consc_pos_3, Q3.12).

Remarks:
See above, under Q3.07

Q3.13 Openness, positively phrased

Question: Here is a list of character traits that which may or may not describe you. Please indicate for each trait whether you think you possess this trait:
Open to new experiences, curious
Response Scale:
7-point scale ranging from '1 - not at all agree' to '7 - totally agree'

Used in variable: open_pos_3

Transformations: The variable open_3 was calculated by subtracting the negatively phrased item on conscientiousness (open_neg_3, Q.3.17) from the positively phrased item on extraversion (open_pos_3, Q3.13).

Remarks:
See above, under Q3.07

Q3.14 Agreeableness, positively phrased

Question: Here is a list of character traits that which may or may not describe you. Please indicate for each trait whether you think you possess this trait:
Agreeable, warm
Response Scale:
7-point scale ranging from '1 - not at all agree' to '7 - totally agree'

Used in variable: agreeable_pos_3
Transformations: The variable agreeable_3 was calculated by subtracting the negatively phrased item on extraversion (agreeable_neg_3, Q.3.10) from the positively phrased item on extraversion (agreeable_pos_3, Q3.14).

Remarks:
See above, under Q3.07

Q3.15 Conscientiousness, negatively phrased

Question: Here is a list of character traits that which may or may not describe you. Please indicate for each trait whether you think you possess this trait:
Chaotic, prone to neglect
Response Scale:
7-point scale ranging from '1 - not at all agree' to '7 - totally agree'

Used in variable: consc_neg_3
Transformations: The variable consc_3 was calculated by subtracting the negatively phrased item on conscientiousness (consc_neg_3, Q.3.15) from the positively phrased item on conscientiousness (consc_pos_3, Q3.12).

Remarks:
See above, under Q3.07

Q3.16 Neuroticism, positively phrased

Question: Here is a list of character traits that which may or may not describe you. Please indicate for each trait whether you think you possess this trait:
Calm, Emotionally Stable
Response Scale:
7-point scale ranging from '1 - not at all agree' to '7 - totally agree'

Used in variable: neurotic_pos_3

Transformations: The variable neurotic_3 was calculated by subtracting the negatively phrased item on neuroticism (variable neurotic_neg_3, Q.3.07) from the positively phrased item on neuroticism (variable neurotic_pos_3, Q3.16).

Remarks:
See above, under Q3.07

Q3.17 Openness, negatively phrased

Question: Here is a list of character traits that which may or may not describe you. Please indicate for each trait whether you think you possess this trait:
Conservative, uncreative
Response Scale:
7-point scale ranging from '1 - not at all agree' to '7 - totally agree'

Used in variable: open_neg_3
Transformations: The variable open_3 was calculated by subtracting the negatively phrased item on conscientiousness (open_neg_3, Q.3.17) from the positively phrased item on extraversion (open_pos_3, Q3.13).

Remarks:
See above, under Q3.07

Q3.18 Rebelliousness

Question: Here is a list of character traits that which may or may not describe you. Please indicate for each trait whether you think you possess this trait:
Rebellious
Response Scale:
7-point scale ranging from '1 - not at all agree' to '7 - totally agree'

Used in variable: rebel_3
Transformation: None

Remarks:
There is no literature on the validity of this item. However, we do not think this question has led to confusion in answering, therefore it is included in the analysis. We advise to treat the results of this item with caution.

Q3.19 Health-mindedness

Question: Here is a list of character traits that which may or may not describe you. Please indicate for each trait whether you think you possess this trait:
Health-minded
Response Scale:
7-point scale ranging from '1 - not at all agree' to '7 - totally agree'

Used in variable: health_minded_3
Transformation: None

Remarks:
There is no literature on the validity of this item. However, we do not think this question has led to confusion in answering, therefore it is included in the analysis. We advise to treat the results of this item with caution.

Q3.20 Independence

Question: Here is a list of character traits that which may or may not describe you. Please indicate for each trait whether you think you possess this trait:
Free, Independent
Response Scale:
7-point scale ranging from '1 - not at all agree' to '7 - totally agree'

Used in variable: independent_3
Transformation: None

Remarks:
There is no literature on the validity of this item. However, we do not think this question has led to confusion in answering, therefore it is included in the analysis. We advise to treat the results of this item with caution.

Q3.21 Perceived Fitbit effect on physical activity

Question: In your opinion, what effect has the Fitbit had on your daily habits? Do you think the Fitbit has helped you...
Increase your level of physical activity?
Response Scale:
strongly disagree, somewhat disagree, somewhat agree, strongly agree

Used in variable: help_active_3
Transformations: Transformed to numerical scale (ordinal): 1 Strongly Disagree 2 Somewhat Disagree 3 Somewhat Agree 4 Strongly Agree
Items Q3.21 – Q3.29 were used to calculate a weighted meisure of perceived effect of the fitbit on the attainment of participants' goals.
perceived_effect_3 is a weighted mean of all answers to questions about the perceived effect of the fitbit in attaining a particular goal (such as getting in shape, losing weight, etcetera; Q3.21–Q3.29), calculated only for those goals that participants have. The perceived effect scores for those goals where participants scored either 3 (somewhat agree to have the goal) or 4 (strongly agree to have the goal) were added and divided by the total number of goals with scores of 3 or 4.

Remarks:
The effect of the Fitbit on fitness goals, like the previous items on the fitness goals themselves (Q1.25-Q1.33), were assessed using 9 items, each assessing a different goal: respondents were asked to indicate on a 4-pt Likert scale (1= strongly disagree, 4 =strongly agree) whether the Fitbit helped them realise their goal to improve sleep / quit smoking / observe nutrition / change nutrition / increase the motivation for physical activity / observe health / increase activity / lose weight. Each item was used as a single item measure of the self-reported effect on the specific goal that was assessed. Research has shown that single-item measures are valid when the attribute (in this case,

agreement) is concrete and singular and the object (in this case, the perceived effect of the Fitbit on the attainment of a fitness goal) is concrete (Bergqvist & Rossiter, 2007). Thus, we can probably conclude that that these single-item self-report measures are a sufficiently valid measure for the purpose of this paper.

Q3.22 Perceived Fitbit effect on sleep quality

Question: In your opinion, what effect has the Fitbit had on your daily habits? Do you think the Fitbit has helped you...
Increase the quality of your sleep?
Response Scale:
strongly disagree, somewhat disagree, somewhat agree, strongly agree

Used in variable: help_sleep_3
Transformations: Transformed to numerical scale (ordinal): 1 Strongly Disagree 2 Somewhat Disagree 3 Somewhat Agree 4 Strongly Agree
Items Q3.21 – Q3.29 were used to calculate a weighted meisure of perceived effect of the fitbit on the attainment of participants' goals.
perceived_effect_3 is a weighted mean of all answers to questions about the perceived effect of the fitbit in attaining a particular goal (such as getting in shape, losing weight, etcetera; Q3.21–Q3.29), calculated only for those goals that participants have. The perceived effect scores for those goals where participants scored either 3 (somewhat agree to have the goal) or 4 (strongly agree to have the goal) were added and divided by the total number of goals with scores of 3 or 4.

Remarks: See above, under Q3.21

Q3.23 Perceived Fitbit effect on smoking

Question: In your opinion, what effect has the Fitbit had on your daily habits? Do you think the Fitbit has helped you...
Quit smoking?
Response Scale:
strongly disagree, somewhat disagree, somewhat agree, strongly agree

Used in variable: help_smoking_3
Transformations: Transformed to numerical scale (ordinal): 1 Strongly Disagree 2 Somewhat Disagree 3 Somewhat Agree 4 Strongly Agree
Items Q3.21 – Q3.29 were used to calculate a weighted meisure of perceived effect of the fitbit on the attainment of participants' goals.
perceived_effect_3 is a weighted mean of all answers to questions about the perceived effect of the fitbit in attaining a particular goal (such as getting in shape, losing weight, etcetera; Q3.21–Q3.29), calculated only for those goals that participants have. The perceived effect scores for those goals where participants scored either 3 (somewhat agree to have the goal) or 4 (strongly agree to have the goal) were added and divided by the total number of goals with scores of 3 or 4.

Remarks: See above, under Q3.21

Q3.24 Perceived Fitbit effect on monitoring diet

Question: In your opinion, what effect has the Fitbit had on your daily habits? Do you think the Fitbit has helped you...
Monitor your diet?
Response Scale:
strongly disagree, somewhat disagree, somewhat agree, strongly agree

Used in variable: help_monitor_diet_3
Transformations: Transformed to numerical scale (ordinal): 1 Strongly Disagree 2 Somewhat Disagree 3 Somewhat Agree 4 Strongly Agree
Items Q3.21 – Q3.29 were used to calculate a weighted meisure of perceived effect of the fitbit on the attainment of participants' goals.
perceived_effect_3 is a weighted mean of all answers to questions about the perceived effect of the fitbit in attaining a particular goal (such as getting in shape, losing weight, etcetera; Q3.21–Q3.29), calculated only for those goals that participants have. The perceived effect scores for those goals where participants scored either 3 (somewhat agree to have the goal) or 4 (strongly agree to have the goal) were added and divided by the total number of goals with scores of 3 or 4.

Remarks: See above, under Q3.21

Q3.25 Perceived Fitbit effect on eating habits

Question: In your opinion, what effect has the Fitbit had on your daily habits? Do you think the Fitbit has helped you...
Change your eating habits?
Response Scale:
strongly disagree, somewhat disagree, somewhat agree, strongly agree

Used in variable: help_eating_habits_3
Transformations: Transformed to numerical scale (ordinal): 1 Strongly Disagree 2 Somewhat Disagree 3 Somewhat Agree 4 Strongly Agree
Items Q3.21 – Q3.29 were used to calculate a weighted meisure of perceived effect of the fitbit on the attainment of participants' goals.
perceived_effect_3 is a weighted mean of all answers to questions about the perceived effect of the fitbit in attaining a particular goal (such as getting in shape, losing weight, etcetera; Q3.21–Q3.29), calculated only for those goals that participants have. The perceived effect scores for those goals where participants scored either 3 (somewhat agree to have the goal) or 4 (strongly agree to have the goal) were added and divided by the total number of goals with scores of 3 or 4.

Remarks: See above, under Q3.21

Q3.26 Perceived Fitbit effect on motivation

Question: In your opinion, what effect has the Fitbit had on your daily habits? Do you think the Fitbit has helped you...
Maintain your motivation for physical activity?
Response Scale:
strongly disagree, somewhat disagree, somewhat agree, strongly agree

Used in variable: help_motivation_3

Transformations: Transformed to numerical scale (ordinal): 1 Strongly Disagree 2 Somewhat Disagree 3 Somewhat Agree 4 Strongly Agree
Items Q3.21 – Q3.29 were used to calculate a weighted meisure of perceived effect of the fitbit on the attainment of participants' goals.
perceived_effect_3 is a weighted mean of all answers to questions about the perceived effect of the fitbit in attaining a particular goal (such as getting in shape, losing weight, etcetera; Q3.21–Q3.29), calculated only for those goals that participants have. The perceived effect scores for those goals where participants scored either 3 (somewhat agree to have the goal) or 4 (strongly agree to have the goal) were added and divided by the total number of goals with scores of 3 or 4.

Remarks: See above, under Q3.21

Q3.27 Perceived Fitbit effect on health

Question: In your opinion, what effect has the Fitbit had on your daily habits? Do you think the Fitbit has helped you...
Keep your health in check?
Response Scale:
strongly disagree, somewhat disagree, somewhat agree, strongly agree

Used in variable: help_health_3
Transformations: Transformed to numerical scale (ordinal): 1 Strongly Disagree 2 Somewhat Disagree 3 Somewhat Agree 4 Strongly Agree
Items Q3.21 – Q3.29 were used to calculate a weighted meisure of perceived effect of the fitbit on the attainment of participants' goals.
perceived_effect_3 is a weighted mean of all answers to questions about the perceived effect of the fitbit in attaining a particular goal (such as getting in shape, losing weight, etcetera; Q3.21–Q3.29), calculated only for those goals that participants have. The perceived effect scores for those goals where participants scored either 3 (somewhat agree to have the goal) or 4 (strongly agree to have the goal) were added and divided by the total number of goals with scores of 3 or 4.

Remarks: See above, under Q3.21

Q3.28 Perceived Fitbit effect on weight loss

Question: In your opinion, what effect has the Fitbit had on your daily habits? Do you think the Fitbit has helped you...
Lose weight?
Response Scale:
strongly disagree, somewhat disagree, somewhat agree, strongly agree

Used in variable: help_weight_3
Transformations: Transformed to numerical scale (ordinal): 1 Strongly Disagree 2 Somewhat Disagree 3 Somewhat Agree 4 Strongly Agree
Items Q3.21 – Q3.29 were used to calculate a weighted meisure of perceived effect of the fitbit on the attainment of participants' goals.
perceived_effect_3 is a weighted mean of all answers to questions about the perceived effect of the fitbit in attaining a particular goal (such as getting in shape, losing weight, etcetera; Q3.21–Q3.29), calculated only for those goals that participants have. The perceived effect scores for those goals

where participants scored either 3 (somewhat agree to have the goal) or 4 (strongly agree to have the goal) were added and divided by the total number of goals with scores of 3 or 4.

Remarks: See above, under Q3.21

Q3.29 Perceived Fitbit effect on physical shape

Question: In your opinion, what effect has the Fitbit had on your daily habits? Do you think the Fitbit has helped you...
Improve your physical shape?
Response Scale:
strongly disagree, somewhat disagree, somewhat agree, strongly agree

Used in variable: help_shape_3
Transformations: Transformed to numerical scale (ordinal): 1 Strongly Disagree 2 Somewhat Disagree 3 Somewhat Agree 4 Strongly Agree
Items Q3.21 – Q3.29 were used to calculate a weighted meisure of perceived effect of the fitbit on the attainment of participants' goals.
perceived_effect_3 is a weighted mean of all answers to questions about the perceived effect of the fitbit in attaining a particular goal (such as getting in shape, losing weight, etcetera; Q3.21–Q3.29), calculated only for those goals that participants have. The perceived effect scores for those goals where participants scored either 3 (somewhat agree to have the goal) or 4 (strongly agree to have the goal) were added and divided by the total number of goals with scores of 3 or 4.

Q3.30 Digital Proficiency

Question: How would you rate your level of usage of the new technologies? Would you call yourself mostly:
Beginner, Regular User, Technophile / Geek
Response scale:
Beginner, Regular User, Technophile / Geek

Used in variable: tech_level_3
Transformations: Transformed to numerical scale (ordinal): 1 Beginner, 2 Regular user, 3 Technophile / Geek

Remarks:
The question is formulated rather broadly. The categories are also rather vague ('novice' or 'regular' in what?) or emotionally loaded ('technophile / geek'). However, the item may still be usable if it the respondents grasped the intention behind the question. As a result, this item might measure something of the 'technological proficiency' of the user (in using the internet, apps, gadgets).
A more valid measure of 'Internet skills' can be found in Deursen, Dijk and Peters (2011). Internet skills are not equivalent to 'technological proficiency', but the 'medium related' factor in this

measure should probably correlate with one considers to be ‘technological proficiency’ in this internet age.
We advise to treat the results of this item with caution.

Q3.31 Exactness

Question: Here is a list of characteristics that you could attribute to the Fitbit. Please indicate whether you think the Fitbit...
Delivers exact information
Response Scale:
7-point scale ranging from '1 - not at all agree' to '7 - totally agree'

Used in variable: eval_exact_3
Transformations: Scores on items Q2.07 – Q2.18 from Questionnaire 2, and items Q3.23 – Q3.40 from Questionnaire 3 were used in a factor analysis, which informed the construction of three scales:
1) Valence (UX_valence), formed by the sum of the items usefulness / practicality (Q2.07), enjoyableness (Q2.08), modernity (Q2.10), fun (Q2.11), credibility (Q3.34), ease of use (Q2.13), level of answering to needs (Q2.15), beauty (Q2.18), and robustness (Q2.19), divided by 9, minus the sum of the items intrusiveness (Q2.09), inconvenience (Q2.14, and cumbersomeness / nuisance (Q2.16), divided by 3.
2) Preciseness (UX_precise), formed by the sum of the items exactness (Q3.31), level of detail (Q3.32), clarity (Q3.33), and reliability (Q2.12), divided by 4, and
3) perceived efficacy (UX_effect), formed by the sum of the items perceived effect on activity increase (Q3.37), perceived effect on health changes (Q3.38), and wellbeing (Q3.39), divided by three.
The results of the factor analysis (PCA) can be found in table 1 (items Q2.07 – Q2.18) and 2 (items Q3.23 – Q3.40) at the end of this multimedia appendix.

Remarks:
The three UX-related factors Valence, Preciseness, and Perceived Efficacy match similar factors in user experience research (e.g. Hassenzahl, 2003). Therefore we may conclude that the results of the factor analysis are valid enough for the purpose of this study.

Q3.32 Detail

Question: Here is a list of characteristics that you could attribute to the Fitbit. Please indicate whether you think the Fitbit...
Delivers detailed information
Response Scale:
7-point scale ranging from '1 - not at all agree' to '7 - totally agree'

Used in variable: eval_detailed_3
Transformations: See above, under Q3.31

Remarks:
See above, under Q3.31

Q3.33 Clarity

Question: Here is a list of characteristics that you could attribute to the Fitbit. Please indicate whether you think the Fitbit...
Delivers clear information
Response Scale:
7-point scale ranging from '1 - not at all agree' to '7 - totally agree'

Used in variable: eval_clear_3
Transformations: See above, under Q3.31

Remarks:
See above, under Q3.31

Q3.34 Credibility

Question: Here is a list of characteristics that you could attribute to the Fitbit. Please indicate whether you think the Fitbit...
Delivers credible information
Response Scale:
7-point scale ranging from '1 - not at all agree' to '7 - totally agree'

Used in variable: eval_credible_3
Transformations: See above, under Q3.31

Remarks:
See above, under Q3.31

Q3.35 Confidence for behavioural decisions

Question: Here is a list of characteristics that you could attribute to the Fitbit. Please indicate whether you think the Fitbit...
Delivers information upon which you can base decisions for healthy behaviour
Response Scale:
7-point scale ranging from '1 - not at all agree' to '7 - totally agree'

Used in variable: eval_useful_3
Transformations: See above, under Q3.31

Remarks:
See above, under Q3.31

Q3.36 Insight in activity

Question: Here is a list of characteristics that you could attribute to the Fitbit. Please indicate whether you think the Fitbit...
Gives insight in your real physical activity
Response Scale:

7-point scale ranging from '1 - not at all agree' to '7 - totally agree'

Used in variable: eval_aware_3
Transformations: See above, under Q3.31

Remarks:
See above, under Q3.31

Q3.37 Enabling increased physical activity

Question: Here is a list of characteristics that you could attribute to the Fitbit. Please indicate whether you think the Fitbit...
Enables you to increase your physical activity
Response Scale:
7-point scale ranging from '1 - not at all agree' to '7 - totally agree'

Used in variable: eval_enables_activity_3
Transformations: See above, under Q3.31

Remarks:
See above, under Q3.31

Q3.38 Enabling improved health

Question: Here is a list of characteristics that you could attribute to the Fitbit. Please indicate whether you think the Fitbit...
Enables you to improve your health
Response Scale:
7-point scale ranging from '1 - not at all agree' to '7 - totally agree'

Used in variable: eval_enables_health_3
Transformations: See above, under Q3.31

Remarks:
See above, under Q3.31

Q3.39 Enabling improved wellbeing

Question: Here is a list of characteristics that you could attribute to the Fitbit. Please indicate whether you think the Fitbit...
Has improved your wellbeing
Response Scale:
7-point scale ranging from '1 - not at all agree' to '7 - totally agree'

Used in variable: eval_wellbeing_3
Transformations: See above, under Q3.31

Remarks:
See above, under Q3.31

Q3.40 Enabling new monitoring habits

Question: Here is a list of characteristics that you could attribute to the Fitbit. Please indicate whether you think the Fitbit...
Has enabled you to develop a habit of checking your stats
Response Scale:
7-point scale ranging from '1 - not at all agree' to '7 - totally agree'

Used in variable: eval_monitor_3
Transformations: See above, under Q3.31

Remarks:
See above, under Q3.31

Literature

Buysse, D. J., Reynolds, C. F., Monk, T. H., Berman, S. R., & Kupfer, D. J. (1989). The Pittsburgh Sleep Quality Index: a new instrument for psychiatric practice and research. *Psychiatry research*, *28*(2), 193-213.

Cappelleri, J. C., Bushmakin, A. G., McDermott, A. M., Sadosky, A. B., Petrie, C. D., & Martin, S. (2009). Psychometric properties of a single-item scale to assess sleep quality among individuals with fibromyalgia. *Health and Quality of Life Outcomes*, *7*(1), 54.

Cooke, P. J., Melchert, T. P., & Connor, K. (2016). Measuring well-being: A review of instruments. *The Counseling Psychologist*, *44*(5), 730-757.

Geurden, B., Wouters, C., Franck, E., Weyler, J., & Ysebaert, D. (2014). Does Documentation in Nursing Records of Nutritional Screening on Admission to Hospital Reflect the Use of Evidence-Based Practice Guidelines for Malnutrition?. *International journal of nursing knowledge*, *25*(1), 43-48.

Gorber, S. C., Schofield-Hurwitz, S., Hardt, J., Levasseur, G., & Tremblay, M. (2009). The accuracy of self-reported smoking: a systematic review of the relationship between self-reported and cotinine-assessed smoking status. *Nicotine & Tobacco Research*, *11*(1), 12-24.

Hassenzahl, M. (2003). The Thing and I: Understanding the Relationship Between User and Product. In: Blythe, M.A. (Ed.) *Funology*. Boston [etc.]: Kluwer Academic Publishers, 31–42.

Jenkins, C. D., Stanton, B. A., Niemcryk, S. J., & Rose, R. M. (1988). A scale for the estimation of sleep problems in clinical research. *Journal of clinical epidemiology*, *41*(4), 313-321.

Jovanović, V. (2016). The validity of the Satisfaction with Life Scale in adolescents and a comparison with single-item life satisfaction measures: a preliminary study. *Quality of Life Research*, *25*(12), 3173-3180.

Kelly, C., Fitzgerald, A., Sentenac, M., Gakewski, J., Molcho, M., & Gabhainn, S. N. (2016). Weight concerns among adolescent boys. *Public health nutrition*, *19*(03), 456-462.

Klesges, R. C., Debon, M., & Ray, J. W. (1995). Are self-reports of smoking rate biased? Evidence from the Second National Health and Nutrition Examination Survey. *Journal of clinical epidemiology*, *48*(10), 1225-1233.

Li, K., Haynie, D., Palla, H., Lipsky, L., Iannotti, R. J., & Simons-Morton, B. (2016). Assessment of adolescent weight status: Similarities and differences between CDC, IOTF, and WHO references. *Preventive medicine*, *87*, 151-154.

Ministère de l'Enseignement Supérieur, de la Recherche et de l'Innovation MESRI (2017). French classification of levels of educational attainment defined by the French statistical commission on vocational training and social advancement. Retrieved on 19/06/2017 from https://publication.enseignementsup-recherche.gouv.fr/eesr/8EN/EESR8EN_Appendix_6-levels_of_educational_attainment.php. Archived by Webcite at http://www.webcitation.org/6rKvsnc0t.

Niedhammer, I., Chastang, J. F., David, S., & Kelleher, C. (2008). The contribution of occupational factors to social inequalities in health: findings from the national French SUMER survey. *Social science & medicine*, *67*(11), 1870-1881.

Patel, A., Rendu, A., Moran, P., Leese, M., Mann, A., & Knapp, M. (2005). A comparison of two methods of collecting economic data in primary care. *Family Practice*, *22*(3), 323-327.

Patrick, D. L., Cheadle, A., Thompson, D. C., Diehr, P., Koepsell, T., & Kinne, S. (1994). The validity of self-reported smoking: a review and meta-analysis. *American journal of public health*, *84*(7), 1086-1093.

Sallis, J. F., & Saelens, B. E. (2000). Assessment of physical activity by self-report: status, limitations, and future directions. *Research quarterly for exercise and sport*, *71*(sup2), 1-14.

Spencer, E. A., Appleby, P. N., Davey, G. K., & Key, T. J. (2002). Validity of self-reported height and weight in 4808 EPIC–Oxford participants. *Public health nutrition*, *5*(04), 561-565.

Storme, M., Tavani, J. L., & Myszkowski, N. (2016). Psychometric properties of the French ten-item personality inventory (TIPI). *Journal of Individual Differences*.

Subar, A. F., Freedman, L. S., Tooze, J. A., Kirkpatrick, S. I., Boushey, C., Neuhouser, M. L., ... & Reedy, J. (2015). Addressing current criticism regarding the value of self-report dietary data. *The Journal of nutrition*, *145*(12), 2639-2645.
