# Supplementary material for: Determinants for Sustained Use of an Activity Tracker: Observational Study
Source: JMIR Mhealth Uhealth. 2017 Oct 30;5(10):e164. doi: 10.2196/mhealth.7311 (PMC5695980; doi:10.2196/mhealth.7311)
Supplement: Multimedia Appendix 2 [file mhealth_v5i10e164_app2.pdf]

Mutlimedia Appendix 2: Principal Component Analysis

Principal component analysis (PCA) was used as an exploratory tool, together with domain knowledge, to reduce the number of user experience variables to a smaller, more manageable and interpretable set. We analyzed the user experience variables at Q2 and Q3 separately, because of the difference in length of experience with the activity tracker.

PCA for items in Q2

We performed a PCA on the 12 user experience variables in Q2, using data from all 575 participants who completed Q2. There was evidence for limited factorability, with all items correlating at least .2 with another item, and 5 items correlating .3 or higher with another item (see Table 1).

Table 1: Correlation matrix for user experience variables in Q2

	Practical	Nice	Intru-sive	Modern	Amusing	Credible	Easy	Embar-assing	Fits my needs	Nuis-ance	Beaut-iful	Robust
Practical		.25	.07	.20	.17	.07	.14	.07	.27	.08	.01	.06
Nice	.25		.00	.19	.21	.13	.20	-.09	.13	-.09	.15	.08
Intrusive	.07	.00		-.09	-.03	-.04	.14	.35	-.04	.33	-.14	-.07
Modern	.20	.19	-.09		.27	.25	.18	-.03	.31	-.04	.10	.04
Amusing	.17	.21	-.03	.27		.11	.16	-.11	.18	-.08	.11	.09
Credible	.07	.13	-.04	.25	.11		.06	-.09	.26	-.09	.12	.21
Easy	.14	.20	.14	18	.16	.06		.08	-.04	.17	-.07	.03
Embarassing	.07	-.09	.35	-.03	-.11	-.09	.08		-.09	.67	-.23	-.10
Fits my needs	.27	.13	-.04	.31	.18	.26	-.04	-.09		-.05	.09	.04
Nuisance	.08	-.09	.33	-.04	-.08	-.09	.17	.67	-.05		-.27	-.10
Beautiful	.01	.15	-.14	.10	.11	.12	-.07	-.23	.09	-.27		.12
Robust	.06	.08	-.07	.04	.09	.21	.03	-.10	.04	-.10	.12	

The first three factors explained 20%, 16%, and 9% of the variance. We decided to maintain only the first factor, because it was the only factor that was clearly interpretable (see Table 2 for factor loadings).

Table 2: Factor loadings for user experience variables in Q2

	**Loadings**		
	**Factor 1**	**Factor 2**	**Factor 3**
**Item**			
Practical	.16	-.39	.02
Nice	.27	-.26	-.42
Intrusive	-.28	-.31	-.04

Modern	.31	-.32	.14
Amusing	.29	-.24	.23
Credible	.29	-.17	.39
Easy	.02	-.36	-.53
Embarassing	-.41	-.37	.17
Needs	.30	-.25	.51
Nuisance	-.41	-.40	.13
Beautiful	.31	.12	-.07
Robust	.20	-.03	.05
% variance explained	20%	16%	9%

The factor loadings were positive for all positive items and negative for the three negative items (*nuisance*, *embarrassing* and *intrusive*). While the factor loadings were relatively low (all below .42), they fitted a consistent pattern, and Cronbach's alpha for this scale was .62. We included the items *easy* and *practical*, even though these items loaded weakly (<.2). We reasoned that the positive sign of these loadings was consistent with the general pattern, and removing them barely influenced alpha.

We created a composite score for *Valence*, using the mean for all positive items, minus the mean for all negative items.

PCA for items in Q3

We also performed a PCA on the 10 user experience in variables in Q3, using data from all 542 participants who completed Q3. There was evidence for reasonable factorability, with all items correlating at least .3 with another item (see Table 3).

Table 3: Correlation matrix for user experience variables in Q3

	Exact	Detailed	Clear	Credible	Useful	Enables aware-ness activity	Incr-eases activity	Improves health	Increases well-being	Enables monitoring
Exact		.79	.69	.75	.50	.45	.41	.40	.38	.35
Detailed	.79		.71	.68	.55	.37	.34	.35	.34	.35
Clear	.69	.71		.65	.51	.46	.35	.30	.32	.39
Credible	.75	.68	.65		.51	.40	.34	.35	.39	.32
Useful	.50	.55	.51	.51		.53	.50	.54	.50	.40
Enables awareness activity	.45	.37	.46	.40	.53		.63	.50	.47	.34
Increases activity	.41	.34	.35	.34	.50	.63		.73	.64	.47
Improves health	.40	.35	.30	.35	.54	.50	.73		.75	.45
Increases well-being	.38	.34	.32	.39	.50	.47	.64	.75		.45
Enables monitoring	.35	.35	.39	.32	.40	.34	.47	.45	.45	

The first three factors explained 53%, 16%, and 7% of variance. The factor loadings (Table 4) showed that the first factor had a similar interpretation (valence) to the first factor extracted for the Q2 items. To avoid conceptual confusion, we did not include this factor. The third factor was not clearly

interpretable, and the explained variance leveled off after the second factor. Hence, we only included the second factor.

Item	Loadings<br>Factor 1	Factor 2	Factor 3
Exact	-.34	-.34	.03
Detailed	-.33	-.38	-.04
Clear	-.32	-.35	-.06
Credible	-.32	-.33	.03
Useful	-.33	.03	.15
Enables awareness activity	-.30	.16	.48
Increases activity	-.32	.38	.14
Improves health	-.32	.41	.03
Increases well-being	-.31	.37	-.05
Enables monitoring	-.26	.17	-.85
% variance explained	53%	16%	9%

Some items loaded positively on the second factor, while others loaded negatively. As these item sets did not seem to be polar opposites conceptually, we based two composite scores on this factor, including those items with loadings larger than .3. The first composite score, *Preciseness*, was formed by the mean of *exact*, *detailed*, *clear*, and *credible* (Cronbach's alpha = .91). The second composite score, *Perceived effect*, was formed by the mean of *increases activity, improves wellbeing* and *improves health* (Cronbach's alpha = .88).
